# Topoisomerase activity is linked to altered nucleosome positioning and transcriptional regulation in the fission yeast *fbp1* gene

**DOI:** 10.1371/journal.pone.0242348

**Published:** 2020-11-12

**Authors:** Ryuta Asada, Satoshi Senmatsu, Ben Montpetit, Kouji Hirota

**Affiliations:** 1 Department of Chemistry, Graduate School of Science, Tokyo Metropolitan University, Hachioji-shi, Tokyo, Japan; 2 Department of Viticulture and Enology, University of California, Davis, Davis, California, United States of America; Southern Illinois University School of Medicine, UNITED STATES

## Abstract

Chromatin structure, including nucleosome positioning, has a fundamental role in transcriptional regulation through influencing protein-DNA interactions. DNA topology is known to influence chromatin structure, and in doing so, can also alter transcription. However, detailed mechanism(s) linking transcriptional regulation events to chromatin structure that is regulated by changes in DNA topology remain to be well defined. Here we demonstrate that nucleosome positioning and transcriptional output from the fission yeast *fbp1* and *prp3* genes are altered by excess topoisomerase activity. Given that lncRNAs (long noncoding RNAs) are transcribed from the *fbp1* upstream region and are important for *fbp1* gene expression, we hypothesized that local changes in DNA topological state caused by topoisomerase activity could alter lncRNA and *fbp1* transcription. In support of this, we found that topoisomerase overexpression caused destabilization of positioned nucleosomes within the *fbp1* promoter region, which was accompanied by aberrant *fbp1* transcription. Similarly, the direct recruitment of topoisomerase, but not a catalytically inactive form, to the promoter region of *fbp1* caused local changes in nucleosome positioning that was also accompanied by altered *fbp1* transcription. These data indicate that changes in DNA topological state induced by topoisomerase activity could lead to altered *fbp1* transcription through modulating nucleosome positioning.

## Introduction

DNA topology, defined by the degree of interwinding of two complementary strands, is an important factor to consider in various DNA-related biological processes, such as DNA repair, replication, and transcription [1]. Positive or negative DNA supercoiling refers to the over- or under- winding of DNA strands. These alterations in DNA topological state are induced by processes that cause DNA strand separation, including transcription [2]. During transcription, positively supercoiled DNA is generated ahead of, and negatively supercoiled DNA behind, RNA polymerase II [3]. To relieve topological perturbations caused by DNA-mediated events, topoisomerases function to break and reseal the DNA to regulate the topological state of the genome [4, 5]. The importance of this is evidenced by the observation that loss of topoisomerases diminishes mRNA transcription in yeast due to the accumulation of positive DNA supercoils [6]. Moreover, transcription arrest occurs in regions over 100 kb from the chromosome ends in yeast, indicating that local DNA topological stress restricts transcription even in eukaryotic linear genomic DNA [7]. In fact, genome-wide analyses of DNA topological states have revealed that negatively supercoiled DNA accumulates at transcription start sites (TSSs), which is closely correlated with transcription levels in fly and human cells [8, 9].

In eukaryotic cells, DNA is packed in chromatin, which composes of an array of histone–DNA complexes called nucleosomes. The position of nucleosomes plays critical roles in the regulation of gene expression and most DNA-related biological processes through limiting the accessibility of DNA binding proteins to DNA [10, 11]. For example, to activate transcription, the nucleosome positioned at the transcription factor binding site would be removed through nucleosome sliding or eviction by ATP-dependent chromatin remodelers to permit transcription factor binding [12, 13]. The loss of chromatin remodelers involved in nucleosome positioning is known to cause cryptic unstable transcription [14], further suggesting the importance of nucleosome positioning for transcriptional regulation.

Nucleosome formation is further impacted by the DNA topological state, as evidenced by the fact that the efficiency of nucleosome formation on topologically constrained DNA is lower than that on relaxed DNA in an *in vitro* reconstitution assay [15]. Moreover, the resolution of DNA topological stress by topoisomerases promotes nucleosome formation on positively supercoiled DNA [16, 17]. Topoisomerase-mediated resolution is also required for SWI/SNF-mediated chromatin remodeling on circular DNA templates [18], highlighting the critical involvement of DNA topology in defining chromatin structure. However, the mechanism(s) by which nucleosome positioning is regulated *via* the topological state of DNA has not been well defined.

The fission yeast *fbp1* gene, encoding fructose-1,6-bisphosphatase, is one of many genes upregulated during glucose starvation stress, which is known to involve regulation of chromatin structure [19–22]. In glucose-rich conditions, gene repression involves nucleosomes positioned around upstream activation sites 1 and 2 (UAS1 and UAS2), the binding sites for two critical transcription factors (TFs) [23]. In the *fbp1* gene promoter region, a lncRNA (long noncoding RNA) referred to as mlonRNA (metabolic stress-induced long noncoding RNA) is also weakly transcribed from a region upstream of the two TF binding sites in the presence of glucose (referred to as mlonRNA-a). Upon glucose starvation, the transcription initiation site shifts towards the TF binding sites, which is preceded by two distinct lncRNA species (mlonRNAs-b and -c) that are transcribed prior to the strong induction of *fbp1* mRNA [24–26]. Coupled with these changes in mlonRNA transcription upon glucose starvation, chromatin at the *fbp1* upstream region converts to an open configuration, with eviction of nucleosomes positioned at UAS1 and UAS2, and TF binding occurs [24, 27, 28].

lncRNA-coupled chromatin modulation raises the possibility that DNA negative supercoils caused by lncRNA-transcription at the *fbp1* upstream region impact chromatin state and transcriptional output. In this study, we have used topoisomerase overexpression and the direct recruitment of topoisomerase to the *fbp1* gene to begin investigating these potential links. We find that topoisomerase activity impacts the maintenance of positioned nucleosomes upstream of *fbp1*, which is important for proper transcriptional control. These findings indicate an importance of DNA supercoiling in the maintenance of positioned nucleosomes that may alter gene expression regulation, including gene loci regulated by lncRNAs.

## Materials and methods

### Fission yeast strains, genetic manipulation, and cell culture

The *Schizosaccharomyces pombe* strains used in this study are listed in S1 Table. Transformation was carried out using the lithium acetate method as described previously [29]. Synthetic dextrose (SD) medium or minimal medium (MM) was used to repress or induce transcription from the *nmt1* promoter, respectively as described previously [30]. This promoter activates in the absence of thiamine, and SD but not MM medium contains thiamine. Cells carrying an expression vector harboring the *nmt1* promoter were pre-cultured in SD medium. After cells had been washed twice with sterile water, they were transferred to MM containing 6% glucose and cultured for 16 h (glucose-rich conditions). Then, cells were transferred to MM containing 0.1% glucose and 3% glycerol (glucose starvation condition). Cells without the expression vector were cultured as described previously [31].

### Primers

The primer sequences used in this study are listed in S2 Table.

### Generation of *top1*- and *top2*-overexpressing strains

Annotated *top1* and reported *top2* coding regions were cloned in the pREP1 vector [32]. For the cloning of the *top1* gene, a 0.4-kb 5′-fragment flanked with a *Sal*I site and a 2-kb 3′-fragment flanked with a *Sal*I site were amplified from cDNA and genomic DNA, respectively. The primer sets p1/p2 and p3/p4 were used for amplification of the 5′ and 3′ fragments, respectively. The full-length *top1* sequence was generated by PCR using these fragments as templates and primers p1 and p4, and was then cloned into the *Sal*I site of the pREP1 vector. For the cloning of the *top2* gene, the *top2* sequence flanked with *Sal*I sites was amplified from genomic DNA using primers p5 and p6 and cloned into the *Sal*I site of the pREP1 vector.

### Construction of *Top2-Gal4-BD* and *Top2-Y781F-Gal4-BD* strains

For the expression of the Gal4-BD fusion protein, we replaced the GFP tag gene with the *GAL4-bd* gene encoding N-terminus Gal4 DNA binding domain of *S*. *cerevisiae* (1–93 amino acid) at *Not*I-*Bgl*II restriction site in the pRGT1 vector [30], which is a derivative of pREP1-based pGFT1 [33]. This vector contains *nmt1* promoter, which strongly induces expression in the absence of thiamine and weakly (~1% of w/o thiamine condition) express in the presence of thiamine [34]. Ser-Gly linker (SGGGG x3) was prepared by annealing of primers p89/p90 and inserted into *Not*I site, generating pREP1-*Gal4-BD* vector. For construction of pREP1-*top2-Gal4-BD* vector, *top2* sequence flanked with *Sal*I sites was amplified from genomic DNA using primer set p5/p91 and cloned into the *Sal*I site in pREP1-*Gal4-BD* vector. For construction of pREP1-*top2Y781F-Gal4-BD* vector, *top2* gene was amplified using mutated primer sets p5/p93 and p92/p91. The resultant PCR products were purified using QIAquick gel extraction kit (Qiagen), then these products were combined by PCR using primer p5/p91 and cloned into *Sal*I site in pREP1-*Gal4-BD* vector. The adjacent UAS2 sequence (-594 to -540) was replaced by the 3xGal4 binding.

### Northern blot analysis and chromatin analysis by MNase digestion of DNA

Northern blot and chromatin analyses by MNase partial digestion were performed as described previously [23]. The probes for northern blotting for *prp3* and Southern blotting for *prp3* were amplified by PCR using the primer sets p9/p10 and p11/p12, respectively.

### ChIP-qPCR

ChIP was performed as described previously [23]. For the quantitation of ChIP DNA samples by qPCR, we used the primer sets p15/p16, p17/p18, and p19/p20 for the detection of UAS1, UAS2, and the *cam1* promoter region, respectively. The *cam1* promoter region was analyzed for normalization.

### Mono-nucleosome mapping

A mono-nucleosome mapping experiment was performed as described previously [35] with some modifications, as briefly described below. Cells were grown to 2.0 × 10^7^/mL and 50 mL of cell culture was crosslinked with 1% formaldehyde for 20 min at room temperature. The reaction was terminated by the addition of glycine to 0.125 M. After centrifugation at 3,500 rpm and 4°C for 1 min, the cells were washed in 1 mL of water, resuspended in 8 mL of pre-incubation solution (20 mM citric acid, 20 mM Na_2_HPO_4_, 40 mM EDTA, 30 mM 2-mercaptoethanol) and incubated at 30°C for 10 min. After centrifugation at 3,500 rpm and 4°C for 5 min, the cells were resuspended with 4 mL of Sorbitol/Tris buffer (1 M sorbitol, 50 mM Tris-HCl pH 7.4) containing 2 mg/mL Zymolyase 100T and 10 mM 2-mercaptoethanol (Nacalai Tesque) and incubated at 30°C for 30 min. Then, the cells were washed in 16 mL of cold Sorbitol/Tris buffer without Zymolyase and 2-mercaptoethanol. After washing, the cells were resuspended in 1.5 mL of cold NP-buffer (1 M sorbitol, 50 mM NaCl, 10 mM Tris-HCl pH 7.4, 5 mM MgCl_2_, 1 mM CaCl_2_, 0.75% NP-40) containing 1 mM 2-mercaptoethanol and protease inhibitor cocktail (Roche). The 500-μL aliquots were treated with 100 U/mL MNase for 30 min at 30°C. In this condition, approximately 70% of whole genome DNA was digested as mono-nucleosomal DNA (S1 Fig). The reaction was quenched by the addition of 130 μL of stop buffer (5% SDS, 100 mM EDTA), followed by incubation at 65°C for 16 h with addition of 10 μL of 20 mg/mL proteinase K (Wako). After the addition of 330 μL of 3 M potassium acetate (pH 5.5), the samples were incubated on ice for 5 min followed by centrifugation at 15,000 rpm and 4°C for 20 min. Supernatants were subjected twice to phenol/chloroform/isoamyl alcohol extraction followed by propanol precipitation. The resultant MNase-digested DNA samples were treated with RNaseA and separated by electrophoresis using a 1.5% TAE agarose gel. The DNA fragments corresponding to mono-nucleosomes (~147 bp) were purified using a gel extraction kit (Qiagen). Tenfold-diluted mono-nucleosomal DNA samples were analyzed by qPCR. For detection at the *fbp1* locus, we used the following primer sets: p21/p22, p23/p24, p25/p26, p27/p28, p29/p30, p31/p32, p33/p34, p35/p36, p37/p38, p39/p40, p41/p42, p43/p44, p45/p46, p47/p48, p49/p50, p51/p52, and p53/p54. For the *prp3* locus, we used the following primer pairs: p55/p56, p57/p58, p59/p60, p61/p62, p63/p64, p65/p66, p67/p68, p69/p70, p71/p72, p73/p74, p75/p76, p77/p78, p79/p80, p81/p82, p83/p84, and p85/p86. These primer sets were designed to amplify 100 ± 33-bp amplicons covering the *fbp1* upstream region and 100 ± 30-bp covering the *prp3* region, each of which overlapped by 16–48 and 19–57 bp with neighboring amplicons, respectively. The primer set for the *cam1* ORF region, p87/p88, was used for normalization.

### RT-PCR

Reverse transcription PCR (RT-PCR) was carried out using SuperScript^™^ III first-strand synthesis system for RT-PCR kit (Invitrogen, CA). Quantification of the expression level of *top1* and *top2* was carried out by qPCR using primer sets: p94/p95 for *top1*, p96/p97 for *top2* and p98/p99 for 18S-rRNA as internal control.

## Results

### Overexpression of topoisomerase causes a growth defect

**Fig 1:**
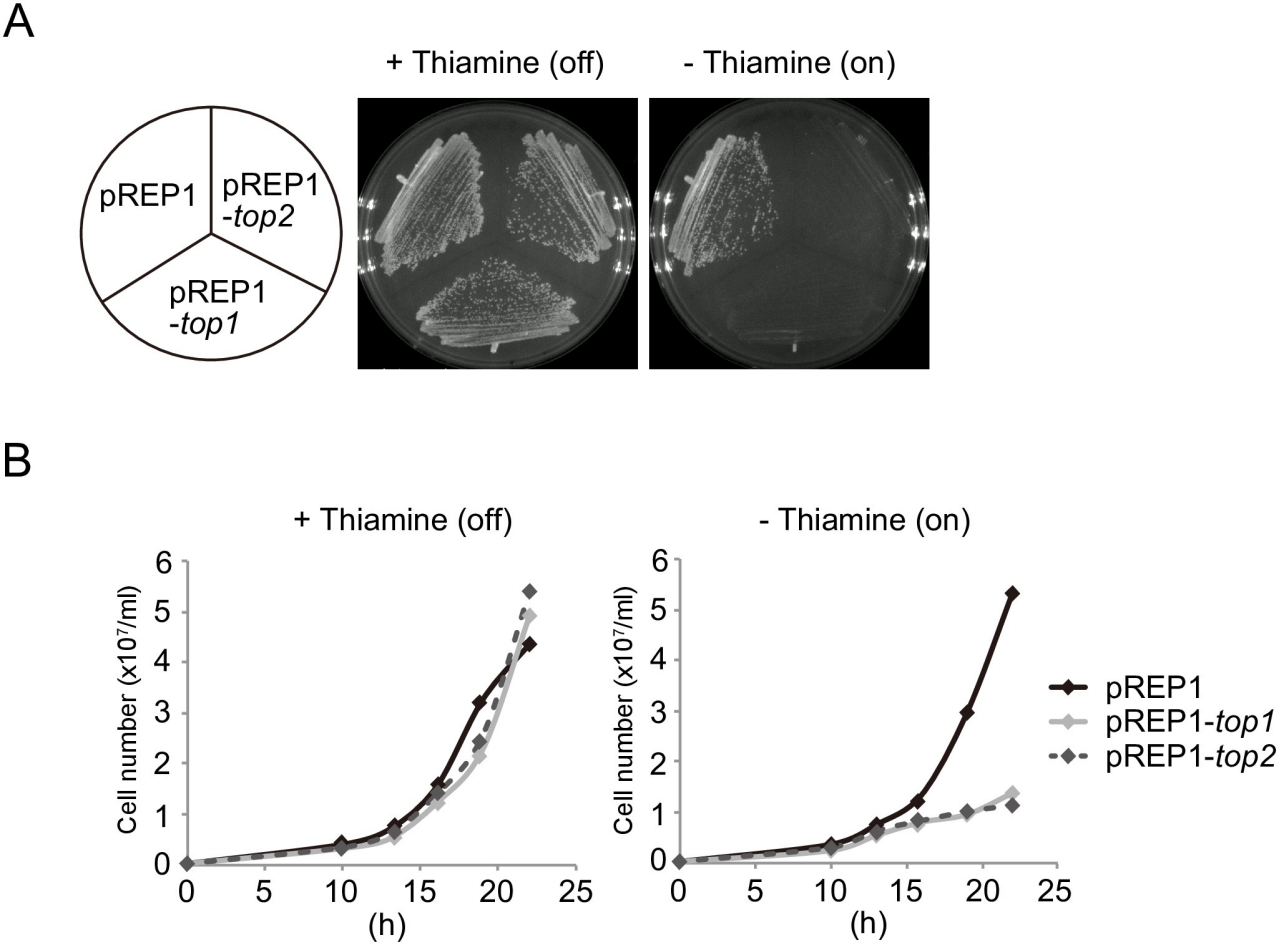
Topoisomerase overexpression is associated with severe growth defect. (A) Single colonies of pREP1-, pREP1-*top1*-, and pREP1-*top2*-carrying cells were streaked to thiamine-containing SD medium (repressive conditions) or thiamine-free MM (inducing conditions) and incubated for 3 days at 30°C. (B) Growth of pREP1-, pREP1-*top1*-, and pREP1-*top2*-carrying cells. Cells were pre-cultured in SD medium and grown to mid-log phase. After washing, the cells were transferred to SD medium or MM and cultured at 30°C.

To examine the role of DNA supercoiling in the regulation of gene expression, we sought to generate cells overexpressing topoisomerase 1 or 2 (Top1 and Top2). Top1 and Top2 resolve both positive and negative supercoils and have overlapping roles in fission yeast [36]. To overexpress these proteins in a controlled manner, Top1 and Top2 were expressed from plasmids using the *nmt1* promoter (pREP1-*top1* and pREP1-*top2*, respectively), which can be conditionally induced by the depletion of thiamine from the medium [34]. Using qPCR, we found that cells carrying these plasmids have higher expression levels of both *top1* (~4 fold) or *top2* (~6 fold) mRNA as compared to control cells in the presence of thiamine (repressive condition), which increased in levels to ~100 fold upon thiamine removal (S2 Fig). Cell proliferation kinetics of pREP1-*top1* and pREP1-*top2* cells were indistinguishable from that of wild-type cells in repressive conditions (Fig 1A), but upon removal of thiamine, cells stopped proliferating after ~16 h of Top1 or Top2 induction (Fig 1A and 1B). These results indicate that overexpression of Top1 or Top2 causes a growth arrest and further suggest a strong toxicity of excess topoisomerase activity in fission yeast.

### Topoisomerase overexpression causes aberrant *fbp1* transcription

**Fig 2:**
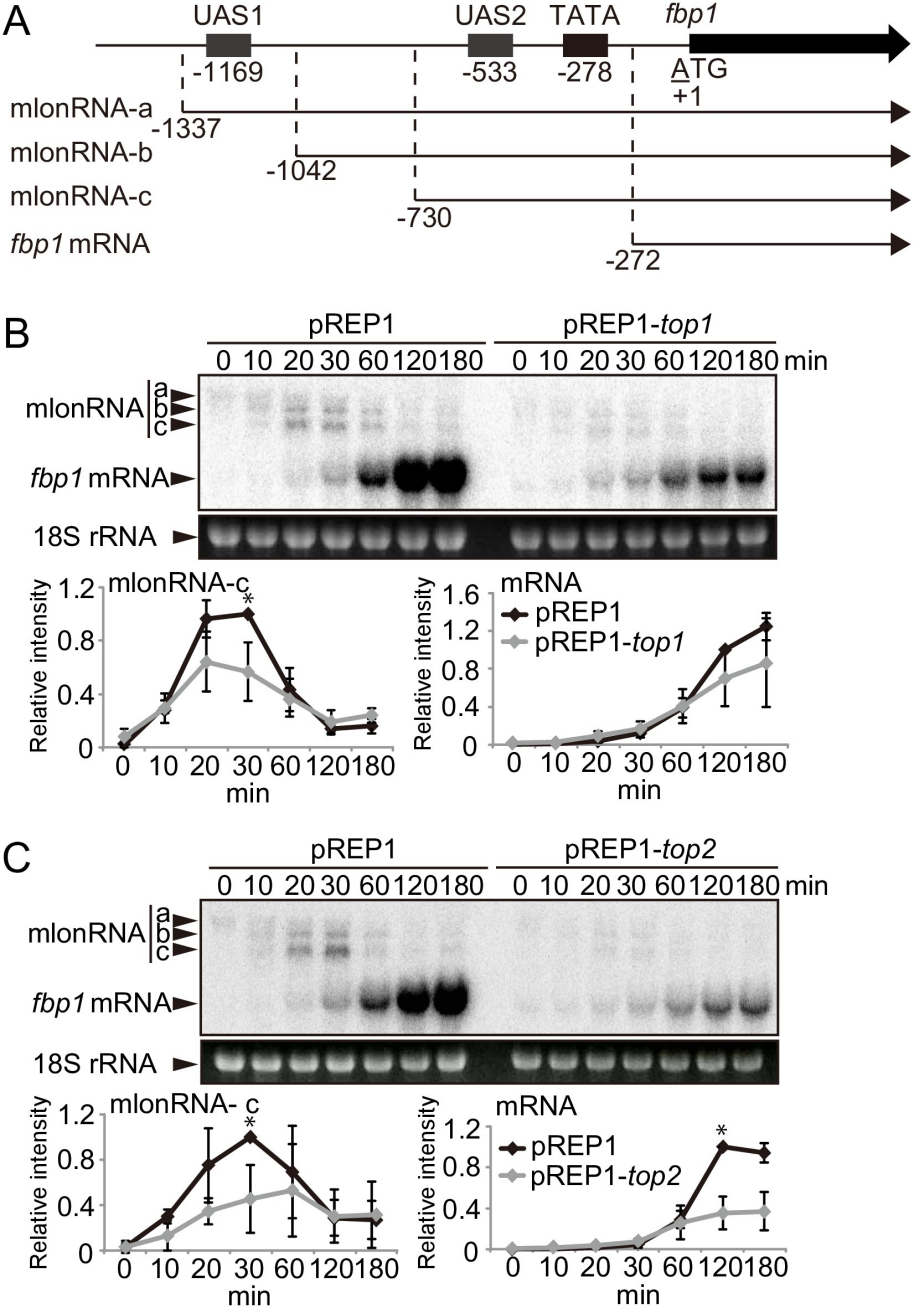
Aberrant *fbp1* transcription by topoisomerase overexpression. (A) Schematic drawing of the region upstream from *fbp1* containing upstream activation sites 1 and 2 (UAS 1 and UAS2). The mlonRNAs transcribed across the *fbp1* upstream region and *fbp1* mRNA are presented. The numbers indicate the transcription initiation site of the *fbp1* transcripts and the distances of UAS1, UAS2, and the TATA box from the first ATG of *fbp1* ORF. (B, C) Transcription of *fbp1* gene during the course of glucose starvation in control cells (carrying the vector [pREP1]), *top1*-overexpressing cells (pREP1-*top1*, B), and *top2*-overexpressing cells (pREP1-*top2*, C). The cells were cultured for 16 h in MM containing 6% glucose (glucose +) and then transferred to MM containing 0.1% glucose and 3% glycerol (glucose −). 18S rRNA was used as a loading control. The band intensities of mlonRNA-c and *fbp1* mRNA were quantitated and normalized for the intensity of 18S rRNA. The error bars indicate standard deviation in three biological replicates. *p*-value was calculated by a Student’s *t*-test: *, *p*<0.05.

We then sought to examine the *fbp1* transcriptional activation profile in Top1 or Top2 overexpressing cells. Given that topoisomerase overexpression strongly inhibits growth, experiments were performed 16 h after Top1 or Top2 induction, which was the earliest timepoint where cultures showed growth differences as compared to control strains (Fig 1B). At this 16 h point of topoisomerase induction in glucose-rich conditions, cells were transferred to glucose starvation conditions to initiate *fbp1* expression. As expected in control cells carrying the empty vector, only mlonRNA-a transcription was observed in glucose-rich conditions, and after glucose starvation, stepwise transcription of mlonRNA-b and -c was detected followed by strong *fbp1* mRNA transcription (Fig 2A–2C, longer exposures of Fig 2B and 2C are shown in S3A and S3B Fig, respectively). In contrast, in the Top1 or Top2 overexpressing cells, low levels of *fbp1* mRNA transcription were already present in glucose-rich repressive conditions (S3A and S3B Fig). Following induction, mlonRNA-b, -c, and *fbp1* mRNA transcriptions were all reduced in the Top1 or Top2 overexpressing cells as compared to control (Fig 2B and 2C). Importantly, the succession of transcription events were still observed in these conditions of topoisomerase overexpression, indicating cells were transcriptionally competent and able to respond to glucose removal at the timepoint tested. These observations indicate that the overexpression of topoisomerases impacts *fbp1* transcriptional control.

### Topoisomerase overexpression alters chromatin structure at the *fbp1* locus

**Fig 3:**
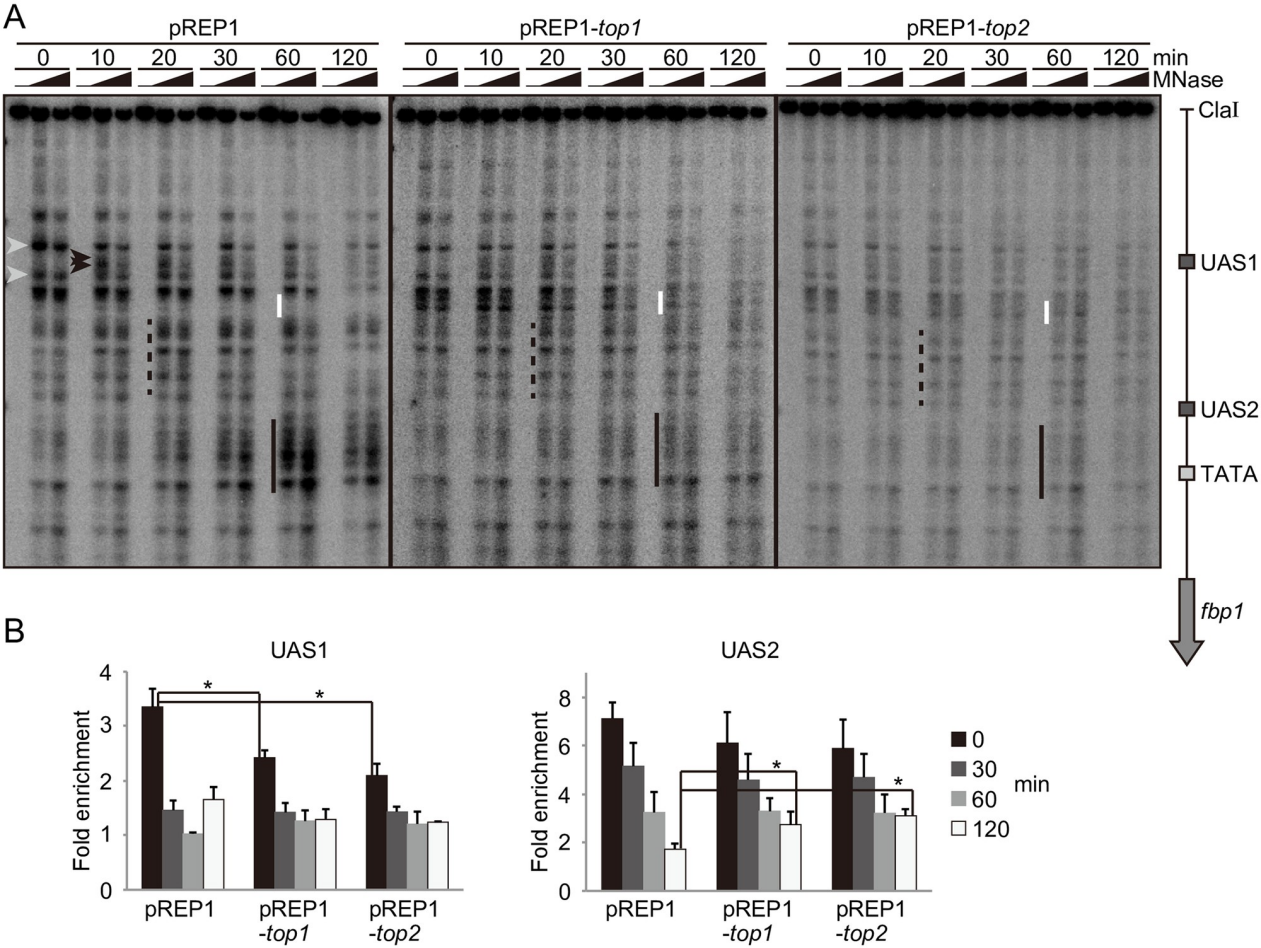
Aberrant chromatin configurations form in the *fbp1* upstream region in topoisomerase-overexpressing cells. (A) Chromatin was analyzed by MNase partial digestion assay in the indicated cells. Cells were cultured as shown in Fig 2. The isolated chromatin DNA was digested with 0, 20, and 50 U/mL MNase. Gray arrowheads represent MNase sensitive sites around UAS1 appeared before glucose starvation. Black arrowheads represent MNase sensitive sites within UAS1. Dotted and black lines represent MNase sensitive sites appeared in glucose starvation around UAS1-UAS2 and TATA box, respectively. White lines represent the region protected from MNase in glucose starvation in control cells. (B) Nucleosome occupancy was analyzed by a ChIP experiment using anti-histone H3 antibody. ChIP signals at UAS1 and UAS2 were normalized for the signal of *cam1* locus as an internal control [39]. The error bars indicate standard deviation from at least two independent experiments. *p*-value was calculated by a Student’s *t*-test: *, *p*<0.05.

Given the central role played by chromatin in *fbp1* transcriptional control [20], we next examined chromatin structure at the *fbp1* upstream region in Top1 or Top2 overexpressing cells. To this end, we employed micrococcal nuclease (MNase) partial digestion of chromatin DNA followed by indirect end-labeling to map nucleosome-free MNase-hypersensitive sites. In control cells under repressive conditions (*e*.*g*. time 0 min), chromatin at UAS1 was protected from MNase digestion, while multiple sensitive sites were present around UAS1 (Fig 3A, gray arrowheads). Following glucose starvation to induce *fbp1* transcription, MNase-sensitive sites appeared within UAS1 as early as 10 minutes after induction in control cells (Fig 3A, black arrowheads). The intensity of the bands corresponding to the UAS1–UAS2 region increased over a 30-minute period (Fig 3A, dotted line). Finally, the intensity of MNase-sensitive sites corresponding to the region around the TATA box increased at 60–120 min after glucose starvation (Fig 3A, black line), while chromatin downstream of UAS1 became protected from MNase (Fig 3A, white line). These observations suggest that stepwise chromatin remodeling is induced upstream of *fbp1* in response to glucose starvation, starting from chromatin containing UAS1/UAS2 and continuing through the TATA box region, while nucleosomes were repositioned at the region downstream of UAS1. In Top1 or Top2 overexpressing cells, MNase-sensitive bands were broad and remained largely unchanged upon glucose starvation in comparison to control cells (Fig 3A). Moreover, the chromatin downstream of UAS1 was not protected from MNase digestion after glucose starvation (Fig 3A, white line). Since pattern of the partial digestion of chromatin DNA was indistinguishable in Top1 or Top2 overexpressing cells and control cells (S4 Fig), overexpression of Top1 or Top2 might have little effect on global chromatin organization, if any. These findings suggest that the overexpression of Top1 or Top2 altered chromatin formation at the *fbp1* gene locus, which was accompanied by an altered transcriptional response (Fig 2 and S3 Fig).

Given the correlation between increased MNase sensitivity and nucleosome eviction from the *fbp1* upstream region [26, 28], we examined alterations in chromatin structure by performing chromatin immunoprecipitation (ChIP) analyses using an anti-histone H3 antibody. Under repressive conditions (0 min), the histone occupancy at UAS1 was significantly reduced by the overexpression of Top1 or Top2 (Fig 3B). After *fbp1* activation, the histone occupancy at UAS1 decreased to the basal level within 30 min of glucose starvation in control cells (Fig 3B). These rapid changes in the UAS1 region seen by anti-histone H3 ChIP are also apparent in cells overexpressing Top1 or Top2 (Fig 3B), but not by MNase digestion assay (Fig 3A). These data suggest that under the conditions of topoisomerase overexpression, nucleosomes around UAS1 are not as well phased and/or have a lower occupancy in glucose rich condition, which may correlate with the low levels of *fbp1* transcription seen in these same cells by northern analysis (Fig 2 and S3 Fig). For UAS2, significant changes in the anti-histone H3 ChIP signals are only apparent at the 120 min activation time point, where residual histone occupancy is detected in Top1 and Top2 overexpressing cells (Fig 3B). These data together with the constant MNase digestion pattern in the UAS2 region of Top1 and Top2 cells, suggest that nucleosomes around UAS2 are also not well phased and/or occupancy is not changing, which may relate to the failure of these cells to strongly induce *fbp1* transcription (Fig 2). Taken together, these findings suggest that topoisomerase overexpression impacts chromatin structure at the *fbp1* upstream region when *fbp1* is repressed and during transcriptional activation.

### Topoisomerase overexpression destabilizes positioned nucleosomes

**Fig 4:**
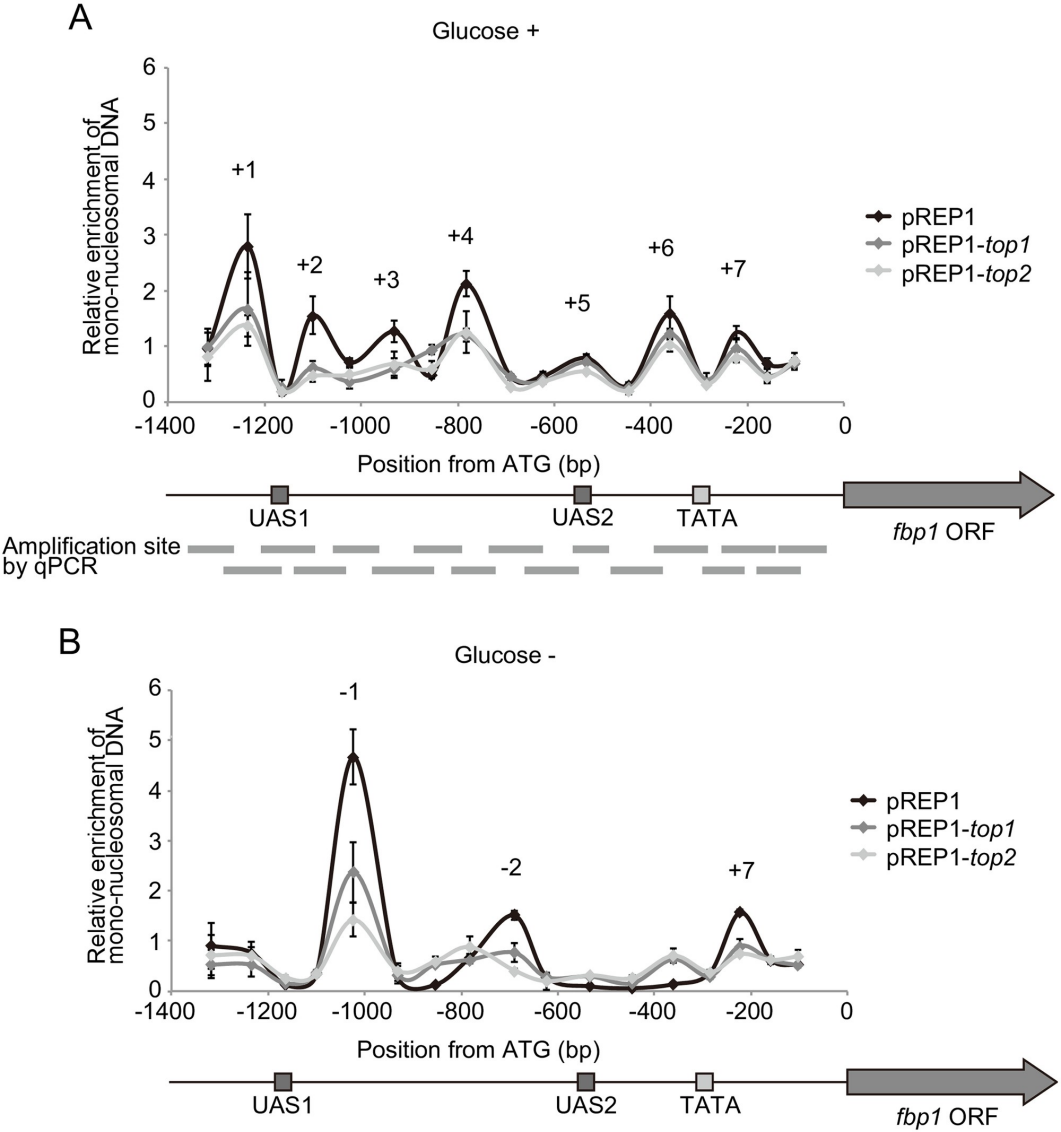
Topoisomerase overexpression destabilizes positioned nucleosomes at the *fbp1* upstream region. (A, B) Mono-nucleosome mapping at the *fbp1* upstream region in control and topoisomerase-overexpressing cells. Cells in glucose-rich and glucose starvation conditions for 120 min (A and B, respectively) were treated with formaldehyde for the crosslinking of nucleosomes and mono-nucleosomes were digested with 100 U/mL MNase. The mono-nucleosomes were analyzed by qPCR using primer sets covering the *fbp1* upstream region. The gray bars represent 17 segments across the *fbp1* upstream region (detected by qPCR). The mono-nucleosome signal at the *cam1* ORF region was used for normalization. Horizontal axis indicates the position from the *fbp1* gene start codon (A of ATG = +1). Error bars indicate standard deviation in at least two biological replicates.

To analyze nucleosome positioning quantitatively, we performed a mono-nucleosome mapping experiment at the *fbp1* upstream region. Mono-nucleosomal DNA was isolated from MNase-digested chromatin and analyzed by qPCR using 17 sets of primers amplifying sequential ~100 bp segments in the *fbp1* upstream region (Fig 4A). In glucose-rich conditions (Glucose +), seven peaks were detected at the *fbp1* upstream region, which are referred to here as +1 to +7 (Fig 4A). This result is consistent with the relative nucleosome positions estimated from a genome-wide study [37], which suggested the presence of seven nucleosomes in this *fbp1* upstream region. Following 120 min of glucose starvation (Glucose −) to induce *fbp1*, two new peaks were detected (referred to here as −1 and −2), while peak +7 remained (Fig 4B). These data show that upon *fbp1* transcriptional activation, nucleosome eviction occurs across most of the upstream region of *fbp1* and nucleosomes are re-positioned at two newly defined sites.

In Top1 or Top2 overexpressing cells in glucose-rich conditions, the peaks of +1 to +4 nucleosomes were decreased in intensity compared with the control cells (Fig 4A). This observation is consistent with the reduced anti-histone H3 ChIP signal at UAS1 in these same conditions (Fig 3B). More specifically, peaks in this region become broader with topoisomerase overexpression, indicating that stable positioning of nucleosomes at precise positions was impaired. In conditions of glucose starvation, Top1 or Top2 overexpressing cells exhibited reduced peaks at −1, −2, and +7 nucleosomes in intensity (Fig 4B). Failure to properly phase the –1 nucleosome in the topoisomerase overexpression cells, with the already weak signal from nucleosomes +1 to +3 in glucose rich condition corresponds well with the lack of changes observed by MNase digestion around the UAS1 region (Fig 3A). Similarly, peaks +5 and +6 do not appear fully eradicated upon glucose removal, which is paralleled by the anti-histone H3 ChIP observed at UAS2 in these same conditions (Fig 3B). Moreover, as detected in glucose rich condition, the -2 peak becomes broader with topoisomerase overexpression, suggesting loss of stable phasing of positioned nucleosome. These findings suggest that topoisomerase overexpression results in defective formation of positioned nucleosome in the *fbp1* upstream region, which is associated with aberrant *fbp1* transcriptional control.

### Topoisomerase overexpression causes aberrant nucleosome positioning at the *prp3* locus

**Fig 5:**
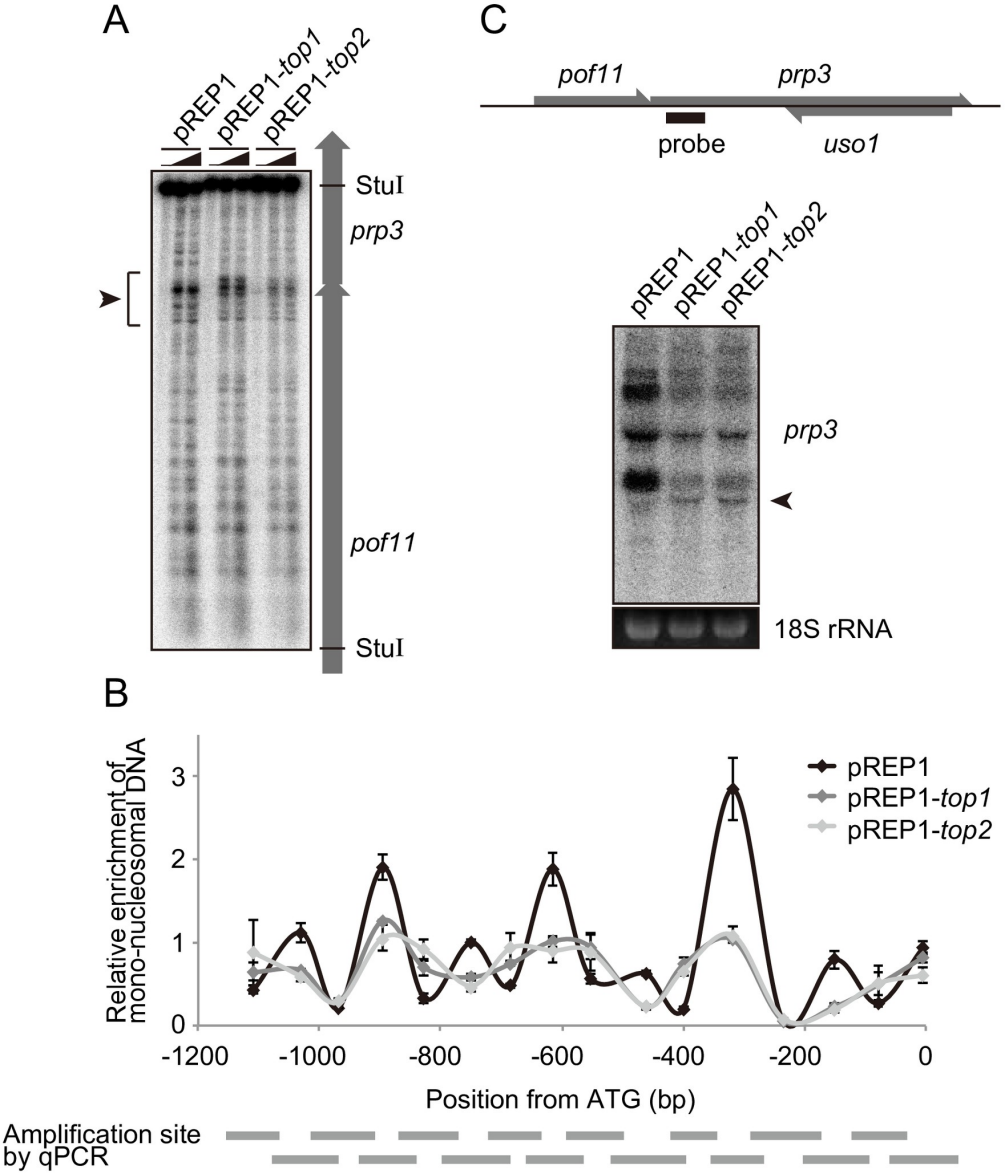
Topoisomerase overexpression destabilizes positioned nucleosomes at the *prp3* promoter. (A) Chromatin state at the *prp3* locus was analyzed by a MNase partial digestion assay in glucose-rich conditions in wild-type and topoisomerase overexpressing cells. The MNase-digested DNA samples used in Fig 3A (0 min) were digested by *Stu*I and subjected to Southern blot analysis. (B) Mono-nucleosome mapping at the *prp3* locus. Purified mono-nucleosomal DNA used in Fig 4 was analyzed by qPCR using primer sets covering the *prp3* upstream region and the 5′ region of the gene body. The gray bars represent 16 segments across the *prp3* upstream region and 5’ region of gene body (detected by qPCR). Horizontal axis indicates the position from the *prp3* gene start codon (A of ATG = +1). The error bars indicate standard deviation in three biological replicates. (C) Representative image of a northern blot showing *prp3* transcript levels in *top1*-overexpressing and *top2*-overexpressing cells as compared to control. Arrowhead indicates a transcript unique to the topoisomerase overexpressing cells. 18S rRNA levels detected by ethidium bromide staining is shown as a loading control.

Topoisomerase overexpression results in a severe growth defect (Fig 1), indicating that chromatin state and chromatin-associated processes may be broadly impacted in these cells. To address whether aberrant nucleosome positioning results from topoisomerase overexpression at other genes, we examined chromatin structure in the promoter region of two constitutively expressed genes which support basic cellular functions, *prp3* and *cam1* [38, 39]. MNase partial digestion assays revealed that chromatin structure around *prp3*, but not the *cam1* gene, was obviously changed by the overexpression of Top1 or Top2 (Fig 5A, S5 Fig). Specifically, chromatin structure within the *prp3* promoter was altered (indicated by bracket and arrowhead in Fig 5A). We thus further examined nucleosome positioning by a mono-nucleosome mapping experiment in the *prp3* promoter region. These assays detected well positioned nucleosomes across the *prp3* region in control cells and peaks that were reduced in intensity and broadened in topoisomerase overexpressing cells (Fig 5B), suggesting aberrant nucleosome positioning. We next analyzed *prp3* transcription in these cell lines and observed at least four distinct transcripts in control cells using a probe within the *prp3* open reading frame (Fig 5C). In the topoisomerase overexpressing cells, the expression level of these transcripts was reduced and an additional short transcript was formed (Fig 5C, arrowhead). These findings indicate that *prp3* transcription is also impacted by topoisomerase overexpression, which is accompanied by changes in both nucleosome positioning in the promoter region of *prp3*. Taken together, data from both the *fbp1* and *prp3* loci are consistent with an ability of excess topoisomerase to destabilize nucleosome positioning in gene promoter regions.

### Forced recruitment of topoisomerase to UAS2 causes local destabilization of nucleosome positioning

**Fig 6:**
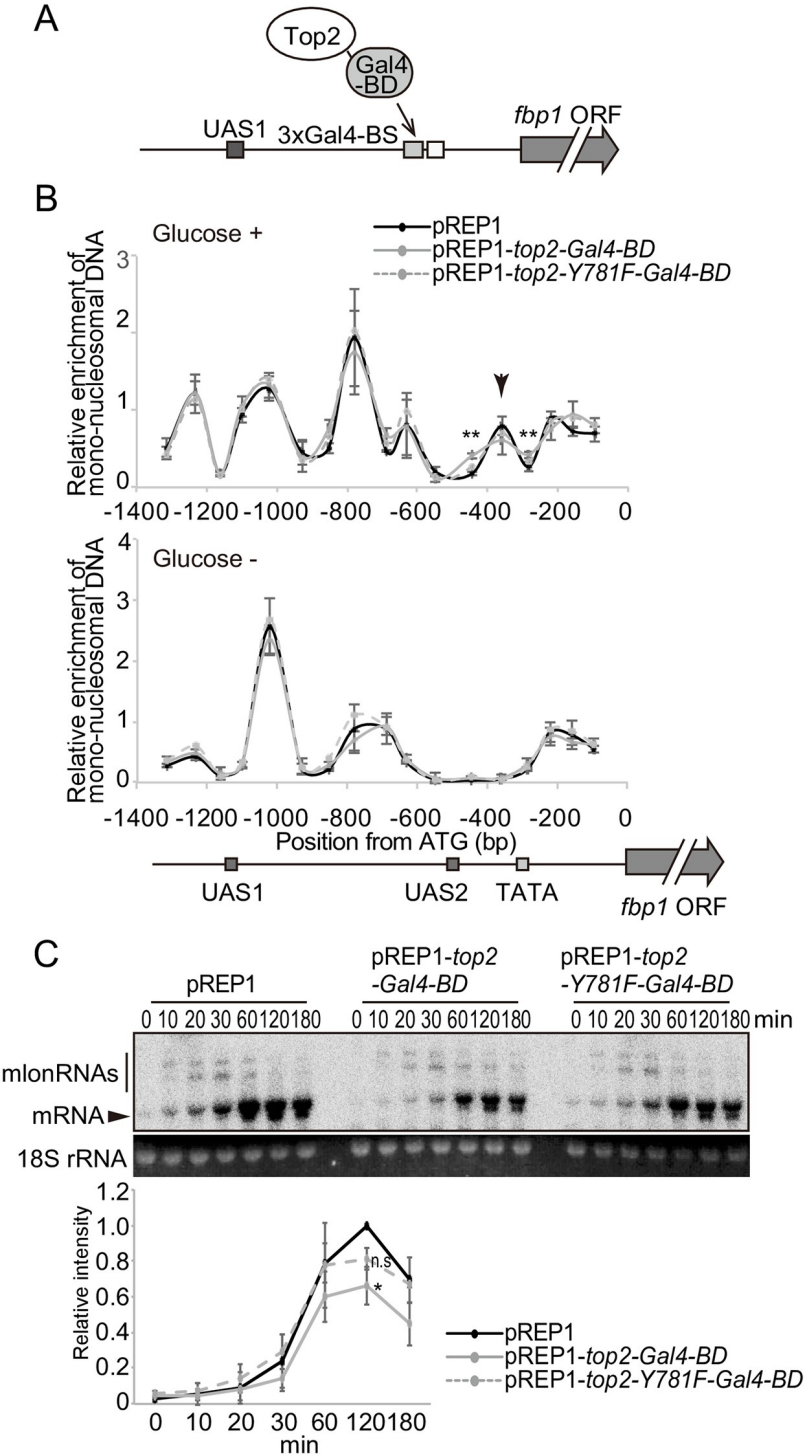
Forced recruitment of topoisomerase to UAS2 causes local destabilization of nucleosomes. (A) Schematic representation of Top2 tethering using Gal4-BD. Three copies of Gal4-binding sequences (BS) were inserted at the upstream adjacent region of UAS2. Gal4-BD was fused to the C-terminus of Top2 expressed from plasmid. (B) Gal4-BD fusion mediated tethering of Top2 to UAS2 causes local destabilization of nucleosome positioning only around UAS2. The mono-nucleosomes were analyzed as in Fig 4. *p*-value was calculated by a Student’s *t*-test: **, *p*<0.01 (C) Transcription of *fbp1* gene during the course of glucose starvation in cells carrying pREP1, pREP1-*top2-GAL4-BD*, or pREP1-*top2*-*Y781F-GAL4-BD*. The cells were cultured in SD (repressed condition). 18S rRNA was used as a loading control. The band intensities of *fbp1* mRNA were quantitated and normalized for the intensity of 18S rRNA. The error bars indicate standard deviation in at least two biological replicates. *p*-value was calculated by a Student’s *t*-test: *, *p*<0.05; n.s, not significant.

The above data indicate a potential role for topoisomerases in regulating nucleosome positioning via the alteration of DNA topology. However, overexpression of Top1 or Top2 caused a growth arrest (Fig 1), and it is thus possible that observed aberrant nucleosome positioning in the topoisomerase overexpressing cells would be secondary caused by the strong toxicity of excess topoisomerases. To specifically examine the impact of topoisomerase on the *fbp1* locus, we employed a Gal4-DNA binding domain (BD) to directly tether Top2 to a region upstream of UAS2 in *fbp1* (Fig 6A) [40, 41]. As a control for the presence of this fusion protein at the promoter, we also used a catalytically defective version of Top2, Top2-Y781F-Gal4-BD. In this construct, a conserved catalytic tyrosine, which attacks a DNA phosphodiester bond during topoisomerase reaction is replaced by phenylalanine [4]. Importantly, experiments using this system were performed in the presence of thiamine, in which the *nmt1* promoter is repressed and expression levels are limited to ~1% the level of the induced condition [34] (S2 Fig). Under this regime, cells expressing Top2-Gal4-BD fusions proliferated with kinetics similar to wild type cells (S6 Fig, left). Notably, Top2-Y781F-Gal4-BD was toxic to cells when induced by thiamine removal, which we presume is due to a dominant negative effect caused by the high levels of protein produced under induction conditions (S6 Fig, right). Importantly, these constructs were only used in the presence of thiamine when the promoter is repressed and the amount of Top2-Gal4-BD produced does not alter cellular growth.

Using this system, we tethered Top2-Gal4-BD to a Gal4-binding sequences inserted upstream of UAS2. In glucose-rich condition (Glucose +), this strain exhibited six peaks by mono-nucleosome profiling, in which peaks +2 and +3 (in Fig 4) were fused into one larger signal (Fig 6B). This change is likely attributable to the insertion of Gal4-binding sequences into this region upstream of UAS2 (Fig 6A). Tethering of Top2, but not Top2-Y781F, in glucose-rich conditions altered one nucleosome downstream of UAS2 leading to a broader peak distribution (Fig 6B, arrowhead), while other nucleosomes in the *fbp1* upstream region were unaffected (Fig 6B). This local changing by the tethering of Top2 was small, but reproducibly observed, and was statistically significant (Double asterisks in Fig 6B, *p*<0.01). This small change is in line with our expectations of this assay, as the topoisomerase is expressed at low levels, it may not always be bound to the promoter (*e*.*g*. displaced by RNA polymerase II), and would need to continually resolve supercoils generated by ongoing mlonRNA transcription. Importantly, cells carrying the Top2-Gal4-BD fusions exhibited no obvious changes in nucleosomes positioning at the *prp3* locus (S7 Fig). During glucose starvation and induction of *fbp1*, the nucleosomes around UAS2 were remodeled and disappeared at the time point tested irrespective of the presence or absence of Top2-Gal4-BD (Fig 6C), but it was observed that the overall transcriptional activation of *fbp1* was impaired by tethered Top2, but not Top2-Y781F (Fig 6C). Taken together, these data indicate that *fbp1* transcriptional control is impacted by local recruitment of enzymatically active Top2 to UAS2.

## Discussion

In the fission yeast, lncRNAs, referred to as mlonRNAs, are transcribed from the *fbp1* gene promoter region and are involved in regulating chromatin remodeling in the *fbp1* upstream region. The production of mlonRNAs is required for the efficient induction of *fbp1* [20, 24]. One potential mechanism for this regulation is through lncRNA-transcription induced negative DNA supercoils in the *fbp1* upstream region that modulate chromatin structure at this locus. In this study, we begin to address the possible role of DNA supercoils in the regulation of *fbp1* by assaying the impact of topoisomerase activity on *fbp1* transcriptional control. We found that over expression of either Top1 or Top2 altered positioned nucleosome in the *fbp1* upstream region in either the repressed or active state (Figs 3 and 4). These changes in chromatin state were accompanied by altered transcriptional outputs that included leaky expression of *fbp1* in the presence of glucose and failure to fully activate transcription upon glucose removal (Fig 2 and S3 Fig). We also observed that excess production of topoisomerase caused destabilization of positioned nucleosome and aberrant transcription in a constitutively expressed gene, *prp3* (Fig 5). These data indicate that DNA topology could broadly function to determine nucleosome positioning *in vivo*.

We found that tethering of Top2, but not an enzymatically inactive point mutant of Top2, caused destabilization of nucleosome positioning (Fig 6). This destabilization happened locally in a limited range near the recruited site. This limited effect was in marked contrast to the drastic effects caused by the overexpression of Top1 or Top2 (Fig 4). This difference might be attributable to the difference of expression level, since there is a ~100 times higher level of the topoisomerase when induced (thiamine −) in comparison to repressive condition (thiamine +) (S2 Fig) [34]. When induced and untethered, the overexpressed Top1 or Top2 might also act at multiple sites in the *fbp1* promoter and upstream region to cause excess resolution of DNA supercoils that impact nucleosome position across the *fbp1* region. Taken together, these experiments indicate that topoisomerase activity is linked to altered nucleosome positioning *in vivo*.

The link between topoisomerase activity and altered nucleosome positioning in *fbp1* and *prp3* upstream regions would suggest that topoisomerases should be enriched in these regions. To address this prediction, we analyzed distribution patterns of Top2 in the upstream region of *fbp1* and *prp3* gene using previously reported ChIP-Seq data for Top2 in glucose rich condition in fission yeast [42]. Through this analysis, strong peaks of Top2 binding within the upstream region of *fbp1* and *prp3* gene are clearly observed, while little Top2 binding was observed in the upstream of *cam1* gene (S8 Fig). It is possible that Top2 at *fbp1* upstream region maintains chromatin states associated with mlonRNA-a transcription. These data further support our findings that topoisomerase activity is linked to the regulation of nucleosome positioning *in vivo* at *fbp1* and *prp3* genes. One outstanding question to address is the relationship and division of labor between the three topoisomerases (*e*.*g*. Top1, Top2 and Top3) in fission yeast. Top1 and Top2 localize at intergenic regions in a similar pattern and have roles in the regulation of transcription through facilitating nucleosome disassembly in fission yeast [43], suggesting Top1 and Top2 might have overlapping roles in the regulation of nucleosome positioning. In contrast, Top3 is enriched at centromere regions, functions in the regulation of CENP-A incorporation [44]. Moreover, Top3 has a suggested role in the resolution of toxic intermediates that arise as a result of dissolution of double Holliday junctions by Rhq1 helicase [45, 46], as evidenced by the observation that lethality of *top3*^-^ cells was completely rescued by the loss of Rqh1 [45]. As such, it is unclear if Top3 would also be involved in regulating nucleosome positioning and will require further investigation.

Stress induced transcription programs must also be effectively repressed once cells adapt to a new environmental condition or the stress is removed. In the case of *fbp1* in fission yeast, it is known that all transcription from this region is immediately repressed when cells are returned from glucose starvation to glucose-rich conditions [47]. One outcome of transcriptional repression may be resolution of negative supercoils at the *fbp1* upstream region, leading to the destabilization and rephasing of nucleosomes to reconstitute a repressive chromatin state. In other words, rapid transcriptional shutoff could directly serve as a signal for the reconstitution to repressive chromatin via the alterations this causes at the level of DNA topology. More broadly, recent studies have revealed that negative supercoils accumulate around TSSs and that the level of DNA supercoils correlates with transcriptional activity in human and fly cells [8, 9]. Since treatment with a topoisomerase inhibitor augments negative supercoiling around TSSs, it is possible that transcription and topoisomerases might commonly counteract each other to modulate DNA topological status at promoter region and transcriptional outputs. In line with this, ChIP-chip analysis in fission yeast demonstrated that the binding of Top1 and Top2 to chromatin are enriched at promoter regions and loss of topoisomerase activity led to an increase in histone occupancy of a target gene promoter region [43]. Similarly in budding yeast, global downregulation of gene expression was observed in *top1*Δ/*top2ts* double mutants, with loss of *PHO5* transcription in this mutant attributed to a nucleosome-remodeling defect [48]. These results parallel the data presented here and suggest that DNA topology may be a conserved mechanism to regulate gene expression through modulating chromatin state within the gene promoter region.

In noncoding regions, such as intergenic and promoter regions, pervasive transcription is commonly observed and in some instances a role of pervasive transcriptions in the regulation of chromosome functions has been highlighted [49–52]. However, in most cases the mechanism underlying such transcription-coupled regulation of chromosome function has not been elucidated. As a result of this study, we suggest DNA topology could be one such determinant of nucleosome positioning *in vivo* that is linked to pervasive transcription and gene expression regulation. We expect that further studies to understand how topoisomerase activity is linked to DNA topology in gene promoter regions, and how this influences lncRNA-transcription, will provide important insights into pervasive transcription and associated biological processes.

## Supporting information

S1 Fig Degree of partial digestion of nucleosomal DNA with MNase in a nono-nucleosome mapping experiment. (A) Representative image showing a DNA sample partially digested with MNase. Partial digestion of nucleosomal DNA with MNase was performed as indicated in Materials and Methods. The sample was run on 1.5% agarose gel at 100 V. (B) Intensities of each band corresponding to mono-, di- and tri-nucleosome were quantified using ImageJ (Plot Profile command). (C) Histogram shows the quantification of intensities of each band corresponding to mono-, di- and tri-nucleosome. Error bars show the standard deviation from three independent experiments.
(PDF)

S2 Fig Expression level of *top1* and *top2* in cells carrying pREP1-*top1* or pREP1-*top2*. mRNA expression level of *top1* and *top2* in cells carrying pREP1-*top1* or pREP1-*top2* in indicated condition was examined by RT-PCR. Level of 18S-rRNA was measured as internal control and used for normalization. Error bars indicate standard deviation in three biological replicates.
(PDF)

S3 Fig *fbp1* transcript are detectable in repressive conditions with topoisomerase overexpression. Representative image of a northern blot showing *fbp1* transcript levels in (A) *top1*-overexpressing cells and (B) *top2*-overexpressing cells as compared to control. Images presented are of a longer exposure of the same northern blot images from Fig 2.
(PDF)

S4 Fig Degree of partial digestion of nucleosomal DNA with MNase in a chromatin analysis by MNase digestion of DNA. (A) Representative image showing a nucleosomal DNA sample partially digested with MNase. Partial digestion of nucleosomal DNA with MNase was performed as indicated in Materials and Methods. The sample was run on 1% agarose gel at 100 V.
(PDF)

S5 Fig Little effect of topoisomerase overexpression at the *cam1* promoter. Chromatin state at the *cam1* locus was analyzed by a MNase partial digestion assay in glucose-rich conditions in wild-type and topoisomerase overexpressing cells. The MNase-digested DNA samples used in Fig 3A (0 min) were digested by NsiI and NcoI and subjected to Southern blot analysis.
(PDF)

S6 Fig Overexpression of Top2-Gal4-BD fusion protein results in a severe growth defect. Single colonies of pREP1, pREP1-*top2-Gal4-BD*, and pREP1-*top2-Y781F-Gal4-BD* carrying cells were streaked to thiamine-containing SD medium or thiamine-free MM and incubated for 3 days at 30°C.
(PDF)

S7 Fig Forced recruitment of topoisomerase to the *fbp1* gene promoter does not change nucleosome positioning at *prp3* promoter. Mono-nucleosome positionings in the cells tethering Top2-Gal4-BD or Top2-Y781F-Gal4-BD to *fbp1* locus were analyzed as in Fig 5B for the *prp3* promoter region. Error bars indicate standard deviation in at least two biological replicates.
(PDF)

S8 Fig Distribution of Top2 at the upstream region of *fbp1* and *prp3*. Chromatin immunoprecipitation (ChIP)-seq data of Top2 in the previous study [42] was analyzed. Genome browser snapshot of Top2 distribution in *fbp1*, *prp3* and *cam1* upstream region was presented. Total genome DNA (WCE) was analyzed as a control.
(PDF)

S1 Table Fission yeast strains used in this study. (PDF)

S2 Table Primers used in this study. (PDF)

S1 Raw images (PDF)
