# Supplementary figures and images for: Topoisomerase activity is linked to altered nucleosome positioning and transcriptional regulation in the fission yeast fbp1 gene
Source: PLoS One. 2020 Nov 12;15(11):e0242348. doi: 10.1371/journal.pone.0242348 (PMC7660550; doi:10.1371/journal.pone.0242348)

**A**

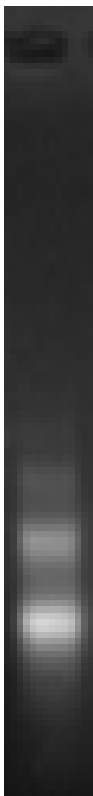

**B**

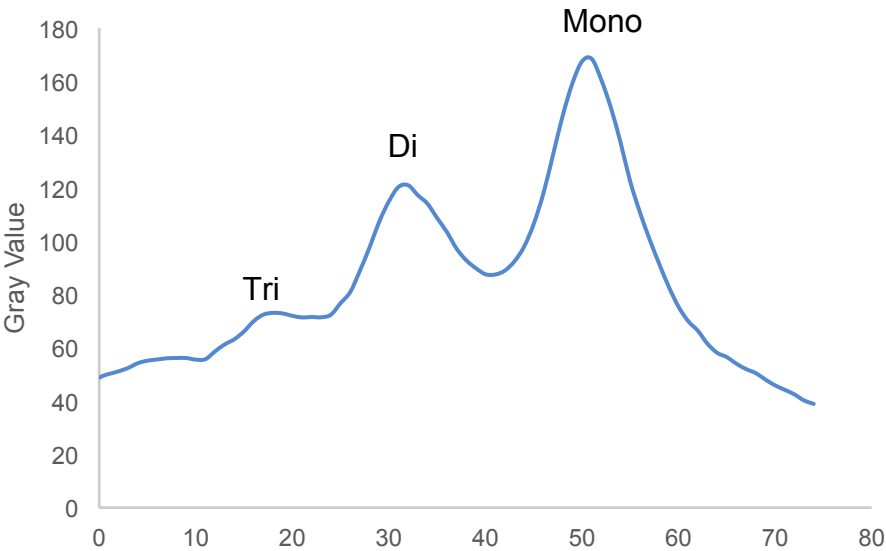

**C**

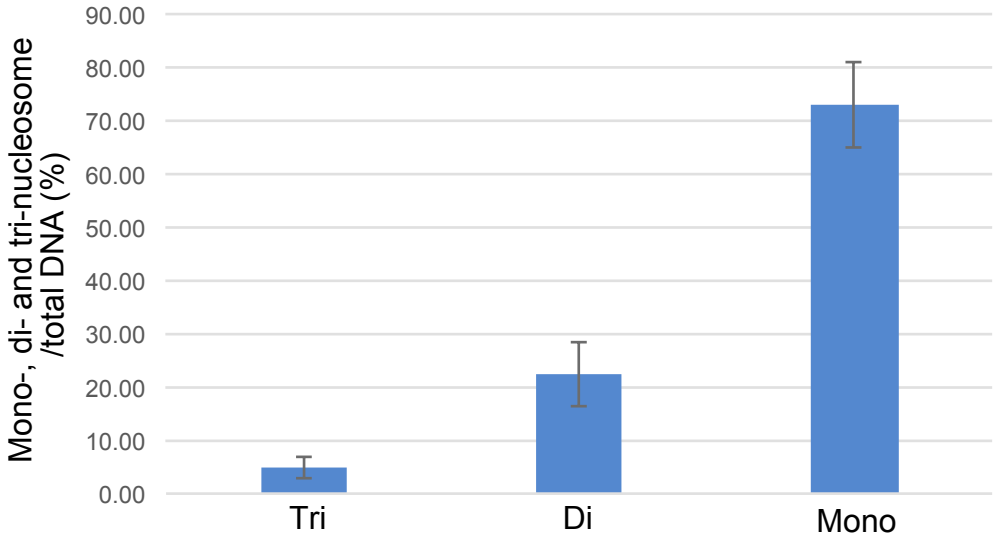

Supplement: S1 Fig — (A) Representative image showing a DNA sample partially digested with MNase. Partial digestion of nucleosomal DNA with MNase was performed as indicated in Materials and Methods. The sample was run on 1.5% agarose gel at 100 V. (B) Intensities of each band corresponding to mono-, di- and tri-nucleosome were quantified using ImageJ (Plot Profile command). (C) Histogram shows the quantification of intensities of each band corresponding to mono-, di- and tri-nucleosome. Error bars show the standard deviation from three independent experiments. (PDF) [file pone.0242348.s001.pdf]

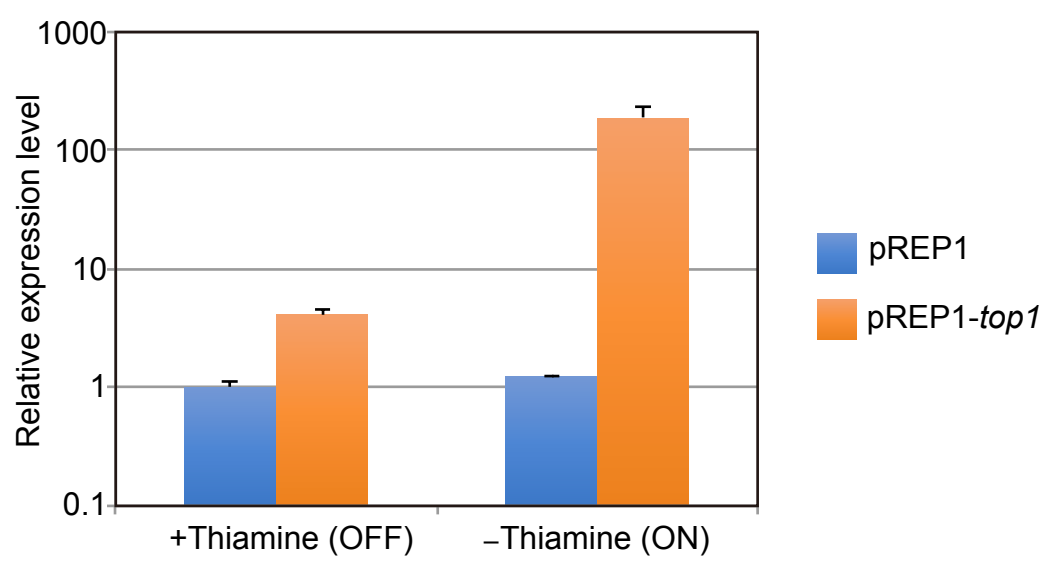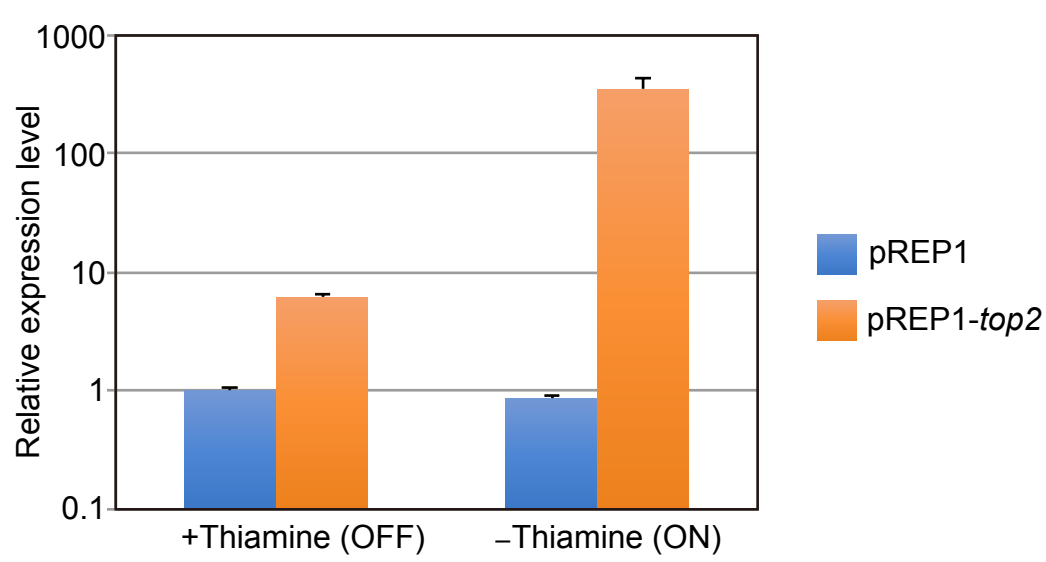

Supplement: S2 Fig — mRNA expression level of top1 and top2 in cells carrying pREP1-top1 or pREP1-top2 in indicated condition was examined by RT-PCR. Level of 18S-rRNA was measured as internal control and used for normalization. Error bars indicate standard deviation in three biological replicates. (PDF) [file pone.0242348.s002.pdf]

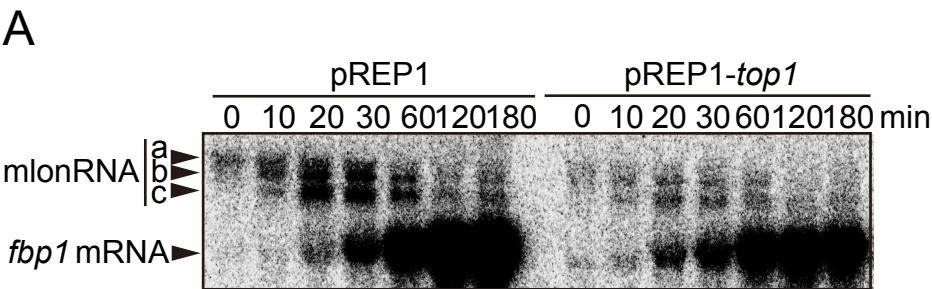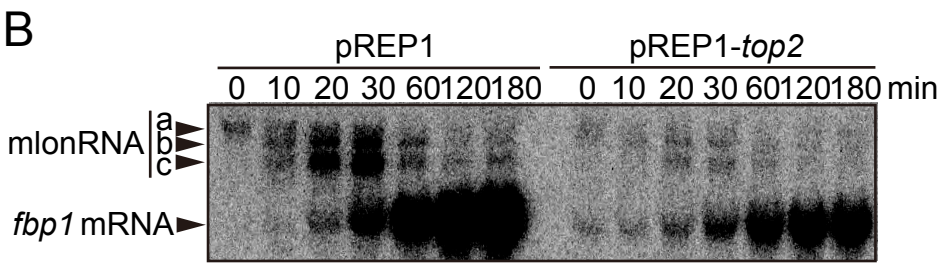

Supplement: S3 Fig — Representative image of a northern blot showing fbp1 transcript levels in (A) top1-overexpressing cells and (B) top2-overexpressing cells as compared to control. Images presented are of a longer exposure of the same northern blot images from Fig 2. (PDF) [file pone.0242348.s003.pdf]

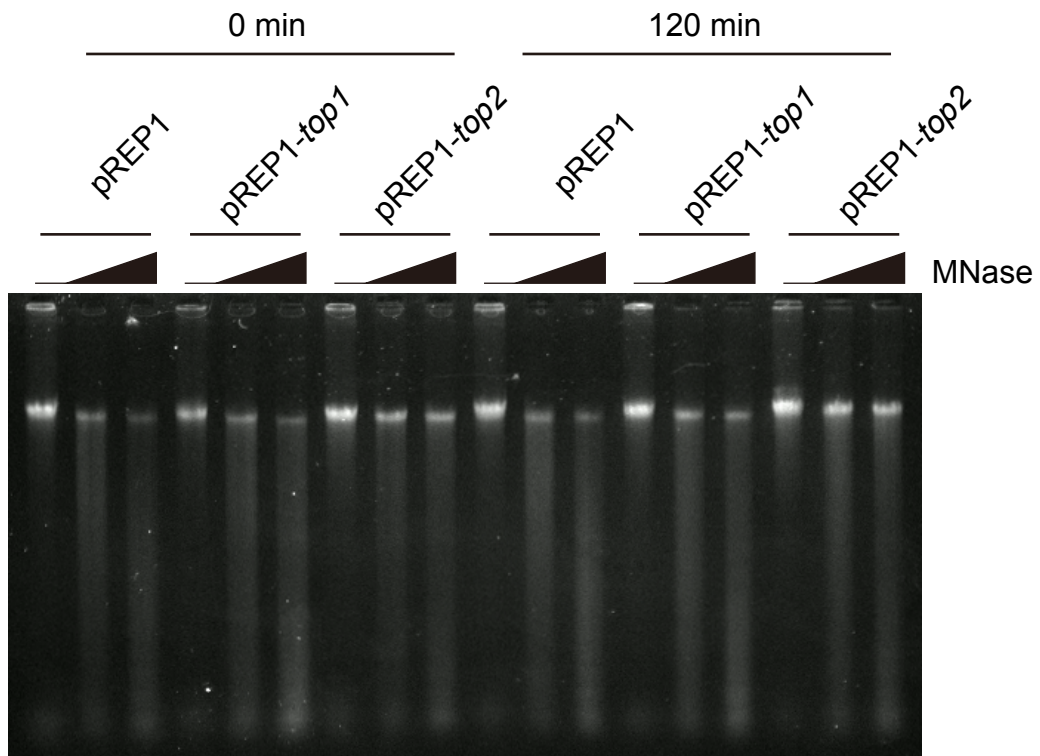

Supplement: S4 Fig — (A) Representative image showing a nucleosomal DNA sample partially digested with MNase. Partial digestion of nucleosomal DNA with MNase was performed as indicated in Materials and Methods. The sample was run on 1% agarose gel at 100 V. (PDF) [file pone.0242348.s004.pdf]

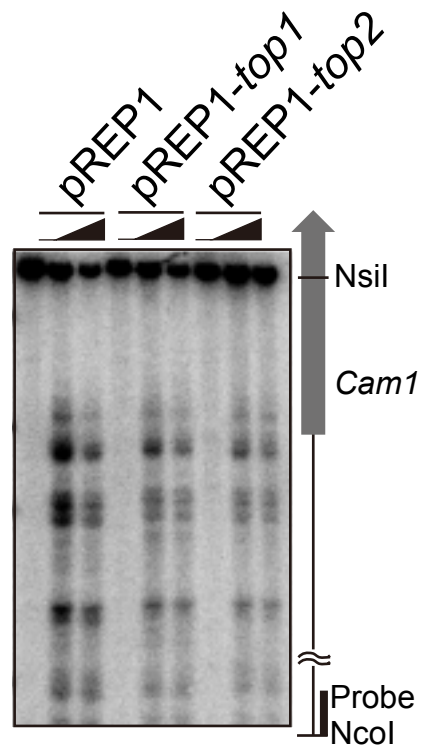

Supplement: S5 Fig — Chromatin state at the cam1 locus was analyzed by a MNase partial digestion assay in glucose-rich conditions in wild-type and topoisomerase overexpressing cells. The MNase-digested DNA samples used in Fig 3A (0 min) were digested by NsiI and NcoI and subjected to Southern blot analysis. (PDF) [file pone.0242348.s005.pdf]

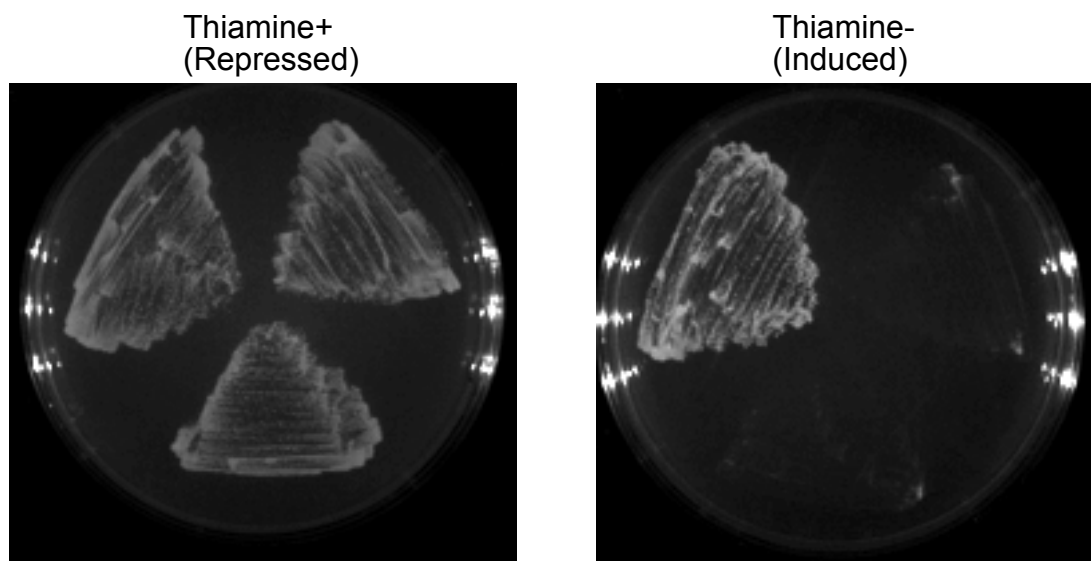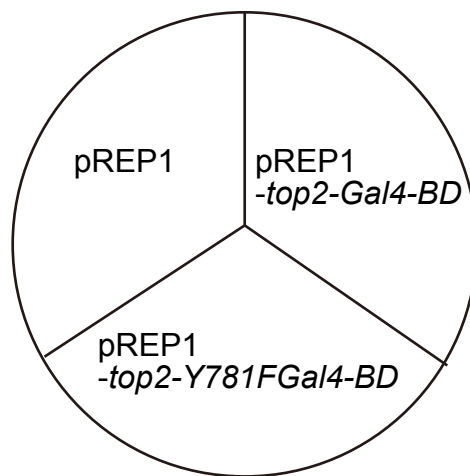

Supplement: S6 Fig — Single colonies of pREP1, pREP1-top2-Gal4-BD, and pREP1-top2-Y781F-Gal4-BD carrying cells were streaked to thiamine-containing SD medium or thiamine-free MM and incubated for 3 days at 30°C. (PDF) [file pone.0242348.s006.pdf]

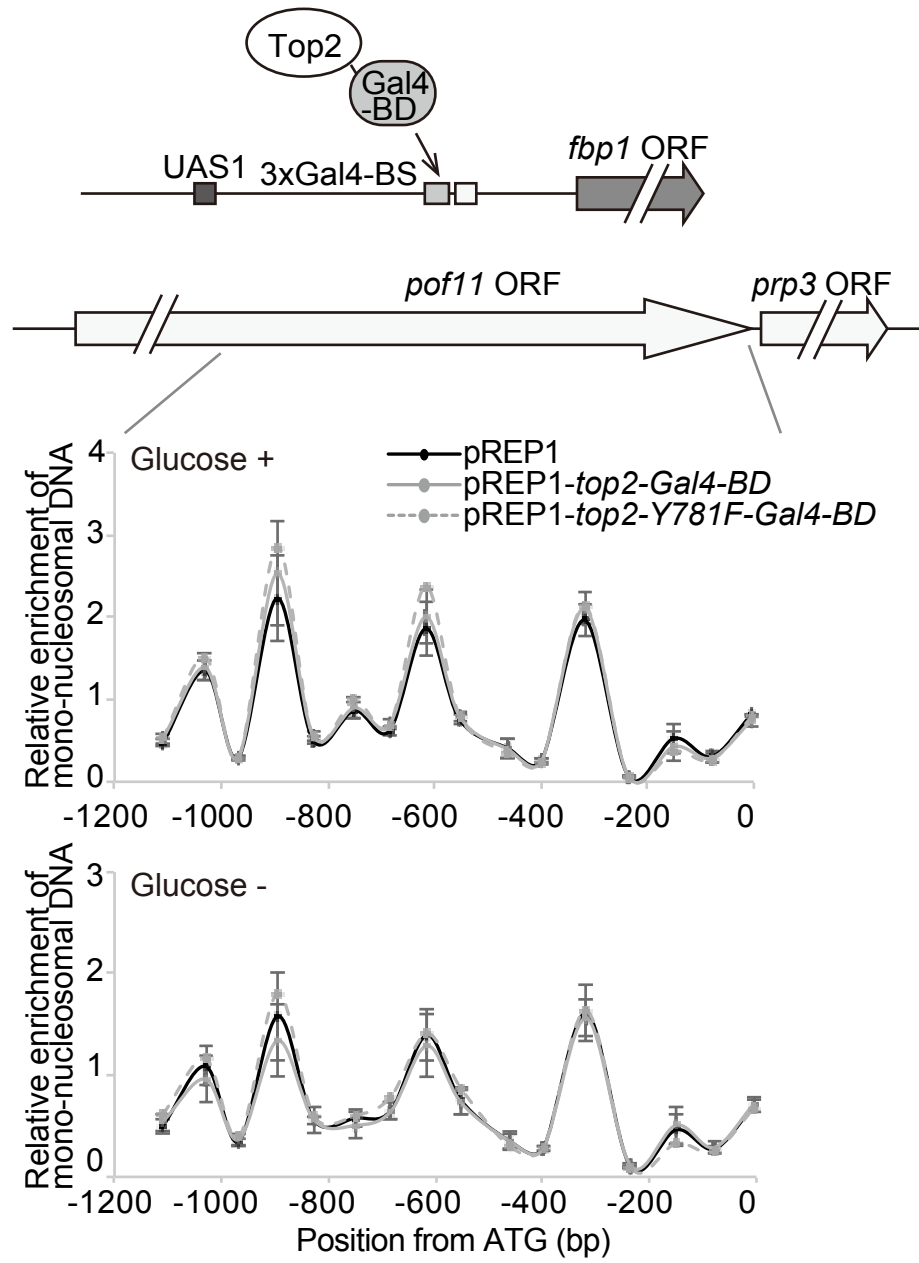

Supplement: S7 Fig — Mono-nucleosome positionings in the cells tethering Top2-Gal4-BD or Top2-Y781F-Gal4-BD to fbp1 locus were analyzed as in Fig 5B for the prp3 promoter region. Error bars indicate standard deviation in at least two biological replicates. (PDF) [file pone.0242348.s007.pdf]

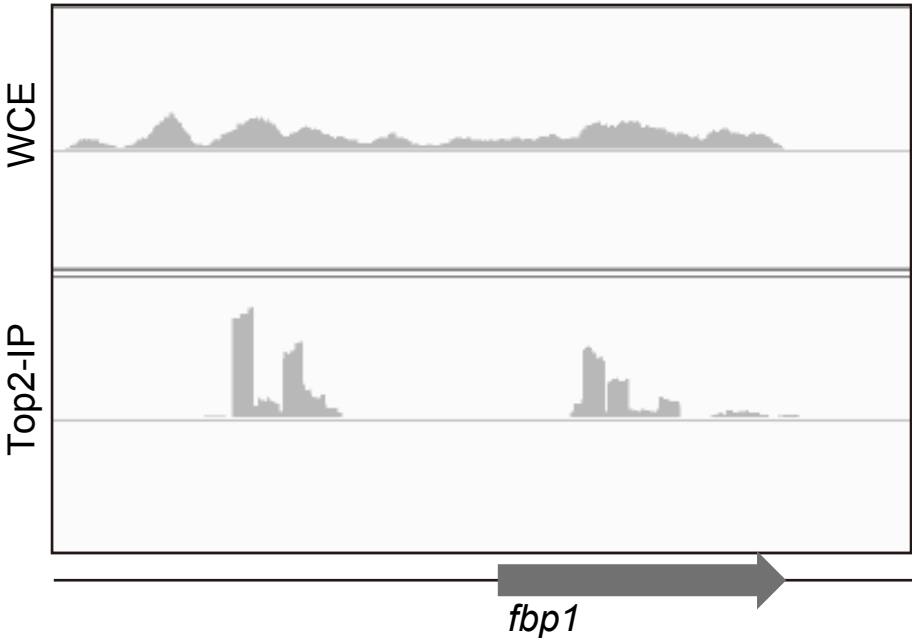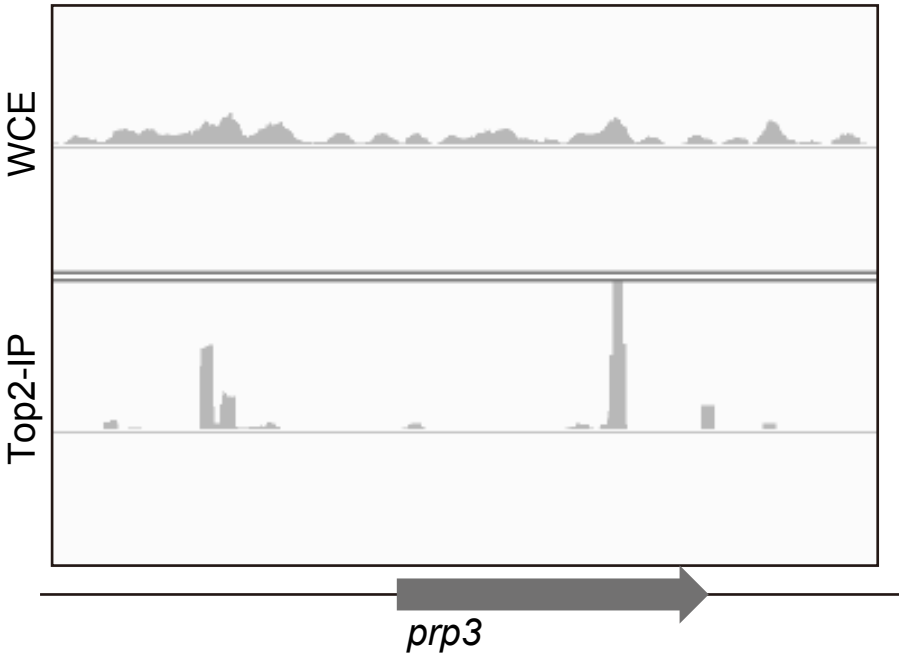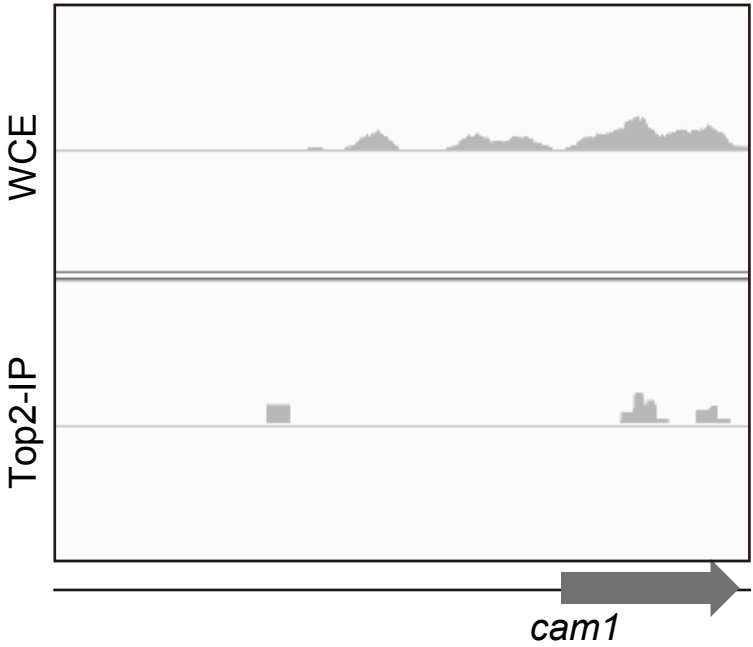

Supplement: S8 Fig — Chromatin immunoprecipitation (ChIP)-seq data of Top2 in the previous study [42] was analyzed. Genome browser snapshot of Top2 distribution in fbp1, prp3 and cam1 upstream region was presented. Total genome DNA (WCE) was analyzed as a control. (PDF) [file pone.0242348.s008.pdf]
