# Supplementary material for: Topoisomerase activity is linked to altered nucleosome positioning and transcriptional regulation in the fission yeast fbp1 gene
Source: PLoS One. 2020 Nov 12;15(11):e0242348. doi: 10.1371/journal.pone.0242348 (PMC7660550; doi:10.1371/journal.pone.0242348)
Supplement: S1 Table — (PDF) [file pone.0242348.s009.pdf]

Supplementary Tables

Table S1. Fission yeast strains used in this study.

**Strain**	**Genotype**
**SPH1**	*h⁻ leu1-32*
**SPH475**	*h⁻ leu1-32 pREP1*
**SPH476**	*h⁻ leu1-32 pREP1-top1*
**SPH477**	*h⁻ leu1-32 pREP1-top2*
**SPH776**	*h⁻ leu1-32 pREP1 fbp1(-594 to -540)::3xGal4 binding site*
**SPH777**	*h⁻ leu1-32 pREP1-top2-Gal4-BD fbp1(-594 to -540)::3xGal4 binding site*
**SPH818**	*h⁻ leu1-32 pREP1-top2Y781F-Gal4-BD fbp1(-594 to -540)::3xGal4 binding site*
