# Supplementary material for: Topoisomerase activity is linked to altered nucleosome positioning and transcriptional regulation in the fission yeast fbp1 gene
Source: PLoS One. 2020 Nov 12;15(11):e0242348. doi: 10.1371/journal.pone.0242348 (PMC7660550; doi:10.1371/journal.pone.0242348)
Supplement: S2 Table — (PDF) [file pone.0242348.s010.pdf]

Table S2. Primers used in this study.

Primer	Sequence
**p1**	AAAAGTCGACAATGTCTTCGTCTGATTCAGATTC
**p2**	TTTAACAGCTGACTTCGCGC
**p3**	GAAGTCAGCTGTTAAAAAAGAGG
**p4**	AAAAGTCGACTTACCACTTCCAATCCGGAG
**p5**	AAAAGTCGACAATGACAGCTTCTGAACAGATAC
**p6**	AAAAGTCGACCTAATCACTCTCATCATAATCGTC
**p9**	CCCAAATCTGAAGGCTTGTTC
**p10**	CCCTAGTTTTCCGTGATTCC
**p11**	GGTCTACGCCCATTTCAG
**p12**	GGAAGCCAACAGATCACG
**p15**	GGGATGAAAACAATCAACCTC
**p16**	GGAATGCAGCAACGAAAATC
**p17**	GGGTGGAATGAGTCCGC
**p18**	GTTCCGCGAATCATAAGCC
**p19**	CGATTTAAACATCGAGTACG
**p20**	GACAGAGCAAATCAAACATG
**p21**	CCTTATTGCTAATAGGAAGG
**p22**	ATAATGACTGGTCATCCAGG

**p23**	GGATGACCAGTCATTATAGA
**p24**	GAAGTGCTAAAGGTGGGTG
**p25**	CTACTAACTACTCCATCCAC
**p26**	CAGCAACGAAAATCAGAAATTG
**p27**	GTACTCATCATCGAATTTCAC
**p28**	CAGAATCAACATAACATGGC
**p29**	CCATGTTATGTTGATTCTGAG
**p30**	GTGTTGTACGTTTTAGAGC
**p31**	GTGTGCTCTAAAACGTACAA
**p32**	CCTATGATTTGATGTCTAGC
**p33**	CGATCAATCATACACAATTGC
**p34**	CTTTCATCACCGACCTCAA
**p35**	GTATGCAATTGAGGTCGGTG
**p36**	GAGAAAATGGCATGGCATG
**p37**	CCATGCCATTTTCTCAGTCAC
**p38**	GGACTCATTCCACCCTATTC
**p39**	ACGTTTTTGCAGTATTATAGG
**p40**	CAGCCTGCTAAACAAATGAC
**p41**	GCAGTATGTCATTTGTTTAGC
**p42**	CTGAGTGATCCGCTTAATTA
**p43**	GCATTTTTAATTAAGCGGATC
**p44**	GTTCCGCGAATCATAAGCC
**p45**	GTTAAAAGGCTTATGATTCGC
**p46**	CAAAAATCAACGAGCCATG
**p47**	AAATACATGGCGCTTCCATC
**p48**	GTCTCTCTCCAGTGTAAAC
**p49**	CAAGGTGAAACGTTTGTTAGG
**p50**	GAGATGATATAATAAGAGACGAG
**p51**	ATCTTCGTCTCTCTCGTCTC
**p52**	GTAGAACTGAGTTGAGCTG
**p53**	CTCACCACTCGGTTTCTAG
**p54**	CCAGGTGAATCCTAGCTG
**p55**	GTCCTGTGTTTGGACTTTTG
**p56**	GCGTCGGTTTTGCCAATC

**p57**	GGGATTGGCAAAACCGAC
**p58**	GGTCCCTTGAAGAAGATATATG
**p59**	GTGTCGTTGTATCCGAAAATTAC
**p60**	CGCAAAACATGCATACAAGC
**p61**	CCCTGCTGAAGCTTGTATG
**p62**	GCTTGCTGTGACAATTAATCC
**p63**	CTTCGAAAACTGGATTAATTGTCAC
**p64**	CGTTGATGAGCATGAATAATGC
**p65**	GCCATTGCATTCGCATTATTC
**p66**	CGGAACTTGCTTCAAATATCC
**p67**	CTTCGGACCTTACAATTAGG
**p68**	GCACAGTGCGGATCAAATC
**p69**	GGTCACGAAGATTTGATCCG
**p70**	CTCGCCAGTGTTGAAATTCC
**p71**	GATGGCACTGTTCGAATTTGG
**p72**	CGTCTGTGGTCAAACTGC
**p73**	GTTCGGTCTGCAGTTTGAC
**p74**	CGCCATTGAATAATAAGTAAGAGAG
**p75**	GACGATGGATTGGATTGTAC
**p76**	CAGGTCTGAGCAAAGGATG
**p77**	CCATCCTTTGCTCAGACC
**p78**	GCAGACATGTTAACAATGGAG
**p79**	GACTCCATTGTTAACATGTCTG
**p80**	GTGTCCTTCAATCTTTAACCTAG
**p81**	GAATGGACCAACTAGGTTAAAG
**p82**	CGTGGTCTAAATATAAACTGTGAAC
**p83**	GCGTTAATGTAAAGTTCACAGTTTA
**p84**	CAAAGCTGGGAATACGAATTTG
**p85**	CACAGTCGTTGTACAAATTCG
**p86**	GGCGAAGAACTTTCCCTC
**p87**	CCCGTAACCTTACAGATGAG
**p88**	CTACACCCAATTCATTGGAC
**p89**	GGCCATCTGGCGGTGGAGGTTCAGGAGGTGGAGGAAGTGGTGGTGGAG
**p90**	GGCCCTCCACCACCACTTCCTCCACCTCCTGAACCTCCACCGCCAGAT

**p91**	AAAAGTCGACCCATCACTCTCATCATAATCGTCTAC
**p92**	TGTATTTAGAAACCTTGAAGCCGATGCATTTTTTC
**p93**	GCTTCAAGGTTTCTAAATACAGCTTTATCACCCTTAG
**P94**	GGATTAGTATGAAGGAGTCG
**p95**	GCACGTCTCTTTCTAATCGG
**p96**	CTAAGGCAGATCAGCAACTC
**p97**	GATTTAGCAGAATCTCCCTC
**p98**	CTCAACTACTTGGATAACCG
**p99**	ATCGAAAGTTGATAGGGCAG
