# Supplementary material for: Topoisomerase activity is linked to altered nucleosome positioning and transcriptional regulation in the fission yeast fbp1 gene
Source: PLoS One. 2020 Nov 12;15(11):e0242348. doi: 10.1371/journal.pone.0242348 (PMC7660550; doi:10.1371/journal.pone.0242348)

Fig. 2B original image

Northern blot

pREP1 — pREP1-*top1*

0 10 20 30 60 120 180 — 0 10 20 30 60 120 180 min

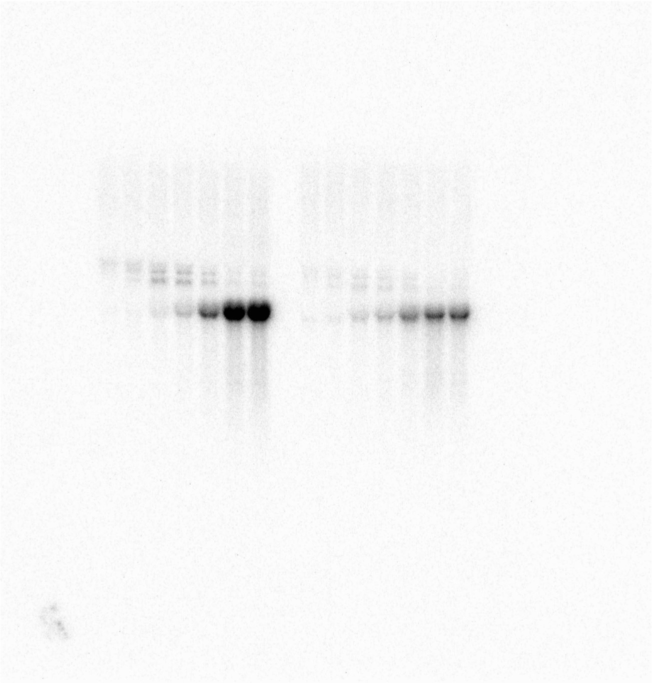

rRNA control

pREP1 — pREP1-*top1*

0 10 20 30 60 120 180 — 0 10 20 30 60 120 180 min

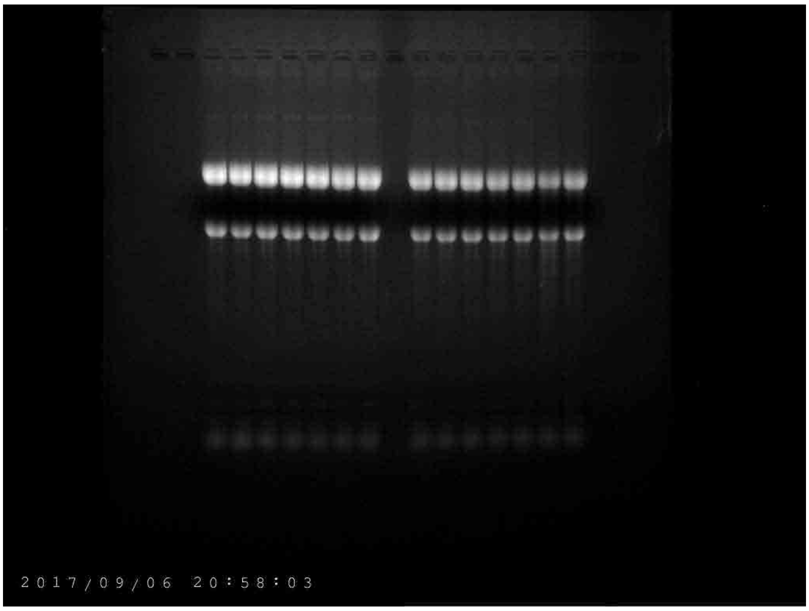

Fig. 2C original image

Northern blot

pREP1 — pREP1-*top2*

0 10 20 30 60 120 180 — 0 10 20 30 60 120 180 min

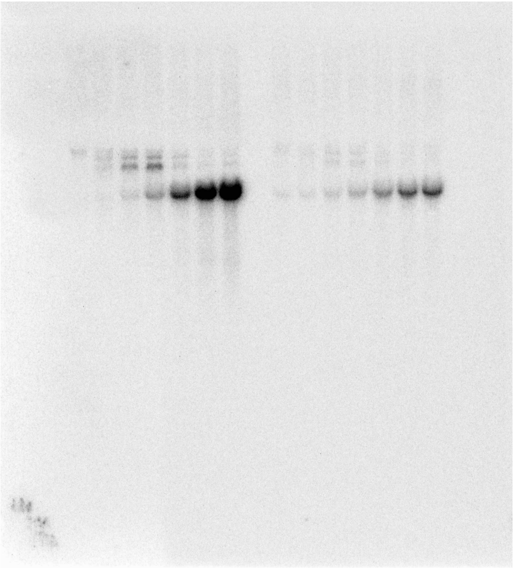

rRNA control

pREP1 — pREP1-*top2*

0 10 20 30 60 120 180 — 0 10 20 30 60 120 180 min

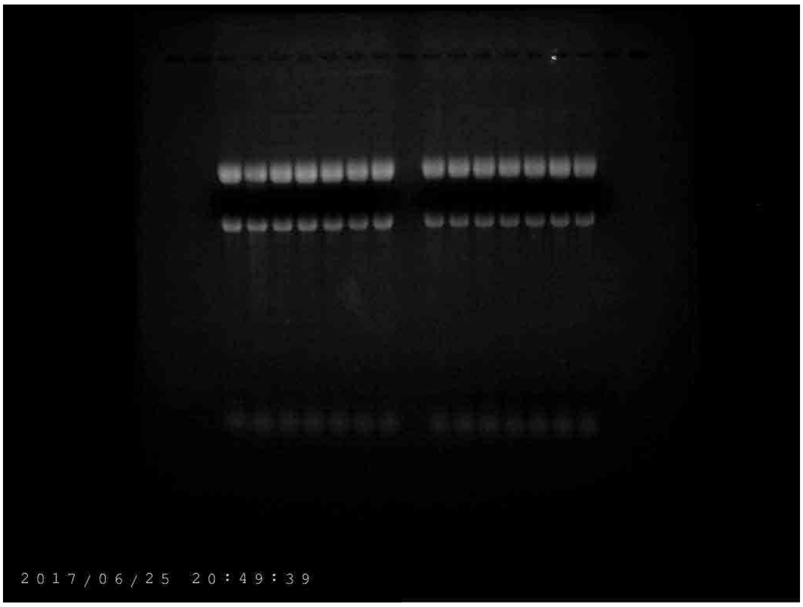

Fig. 3 original image

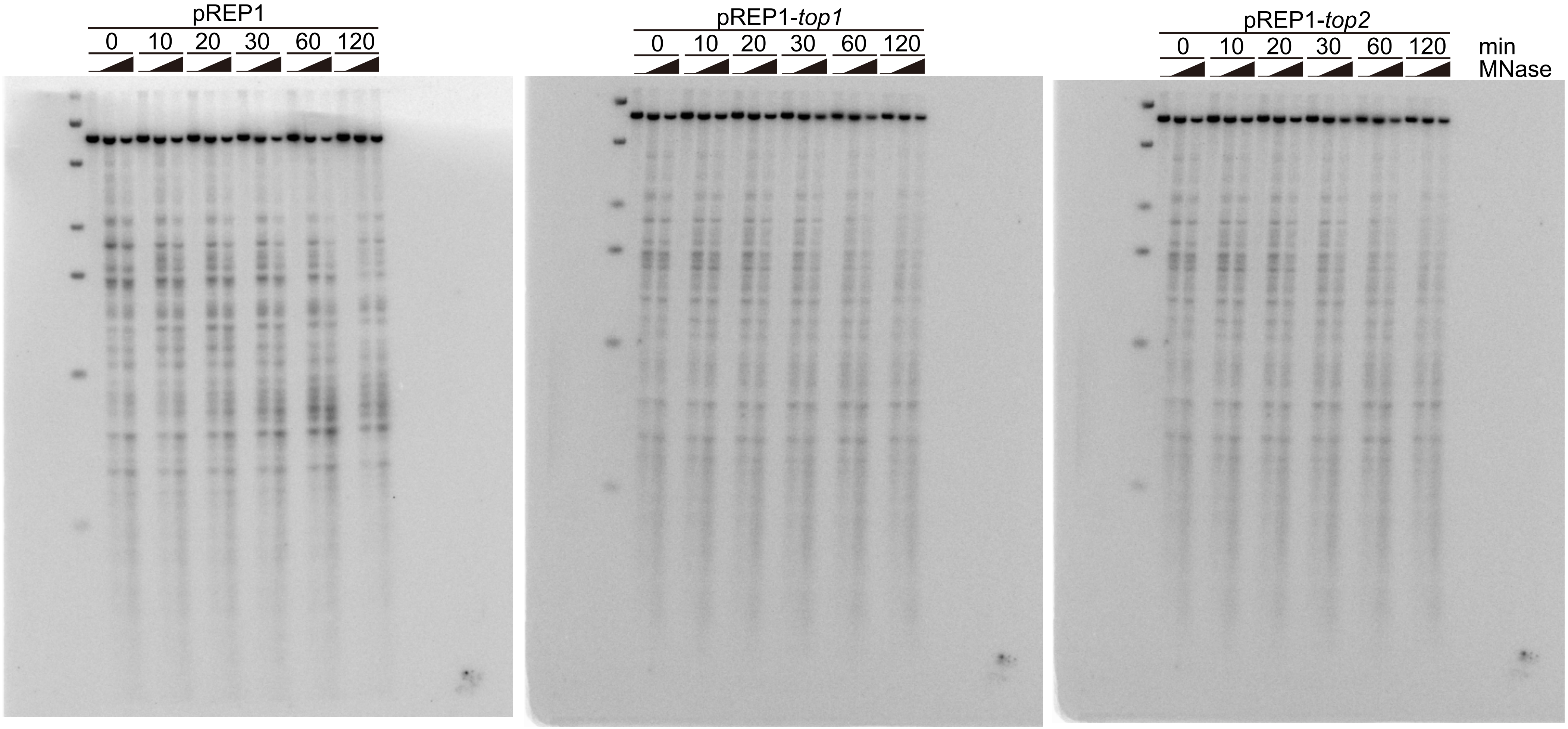

Fig. 5 original image

Fig. 5A original image

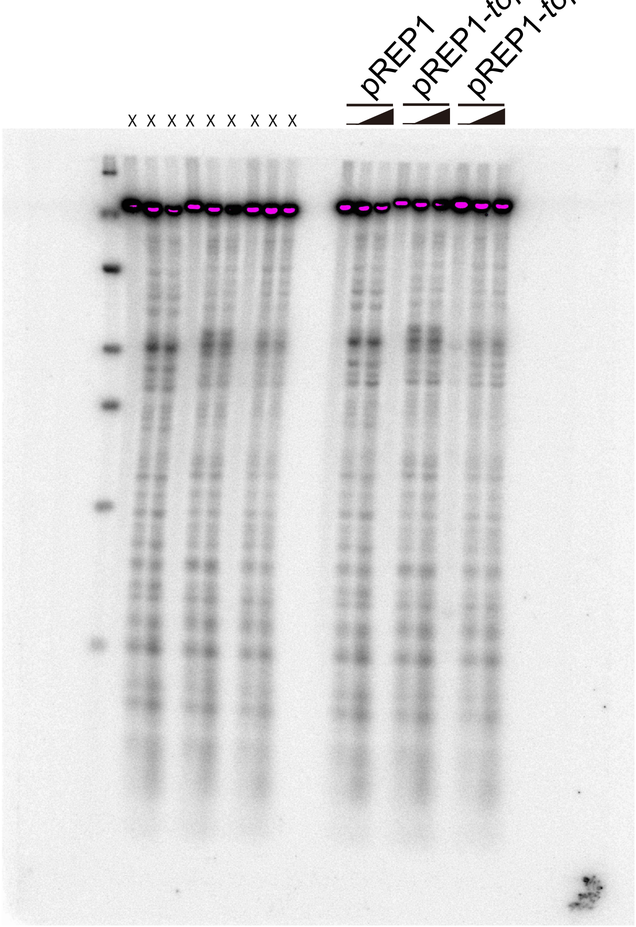

Fig. 5C original image

Northern blot

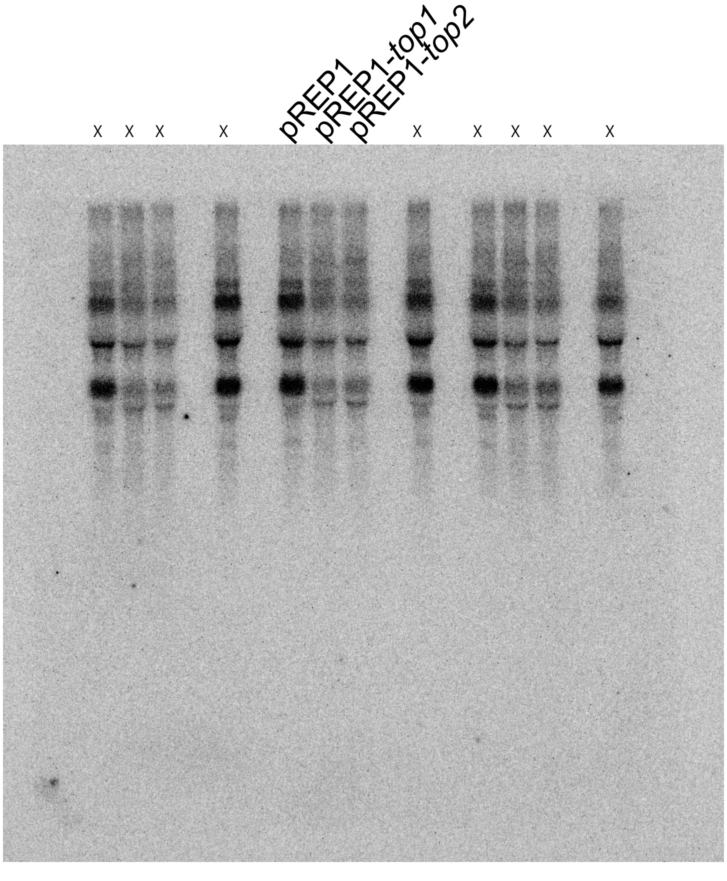

rRNA control

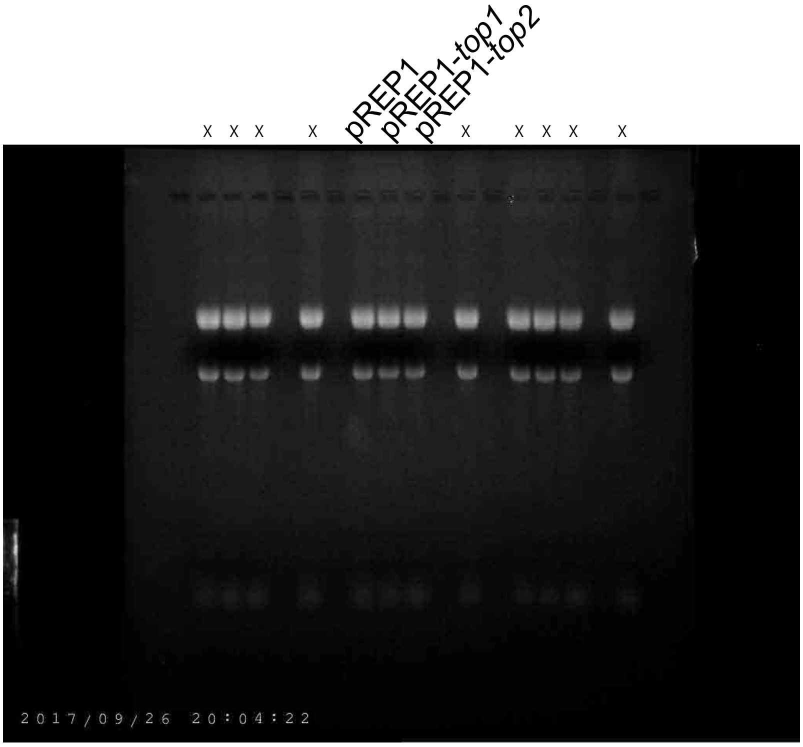

uncropped image used in Fig.6

fbp1 transcripts

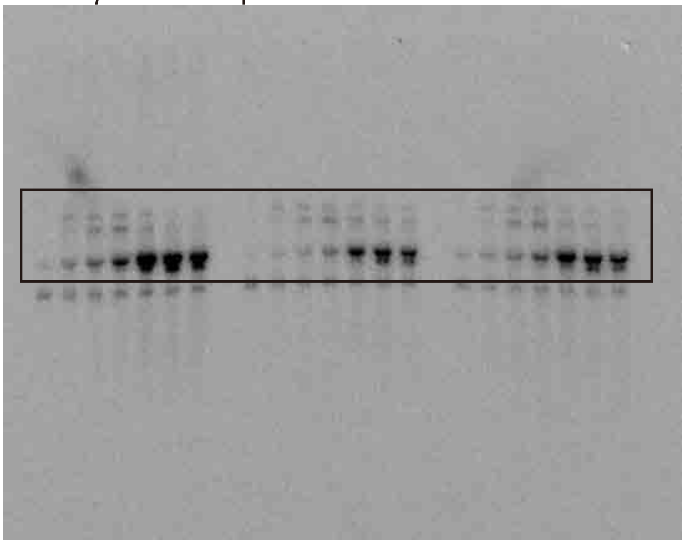

18S rRNA

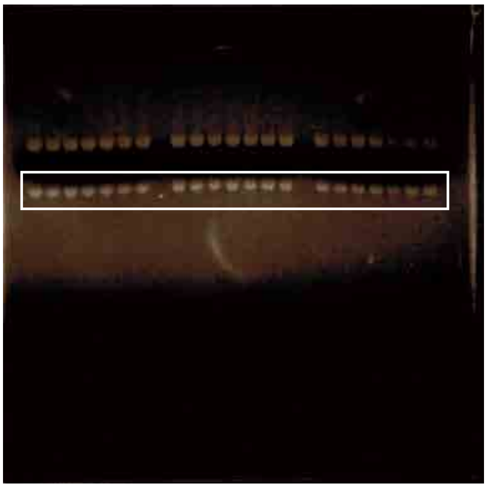

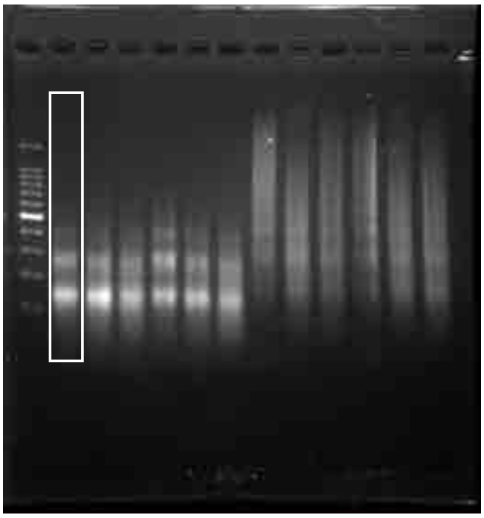

uncropped image used in Fig.S4

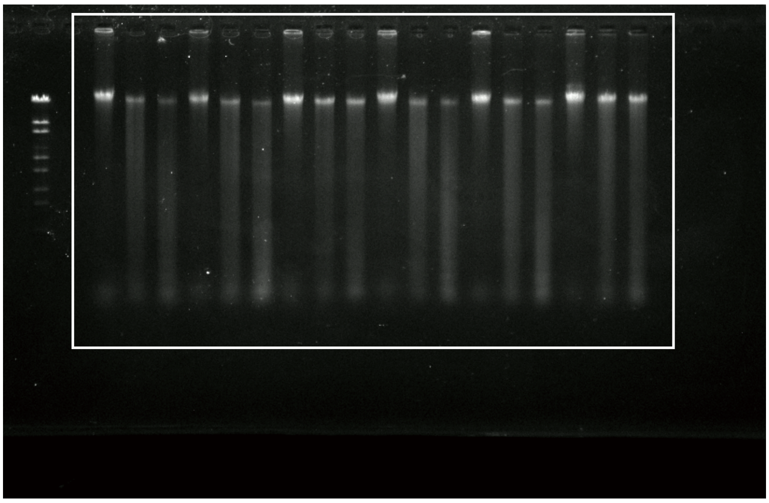

uncropped image used in Fig.S5

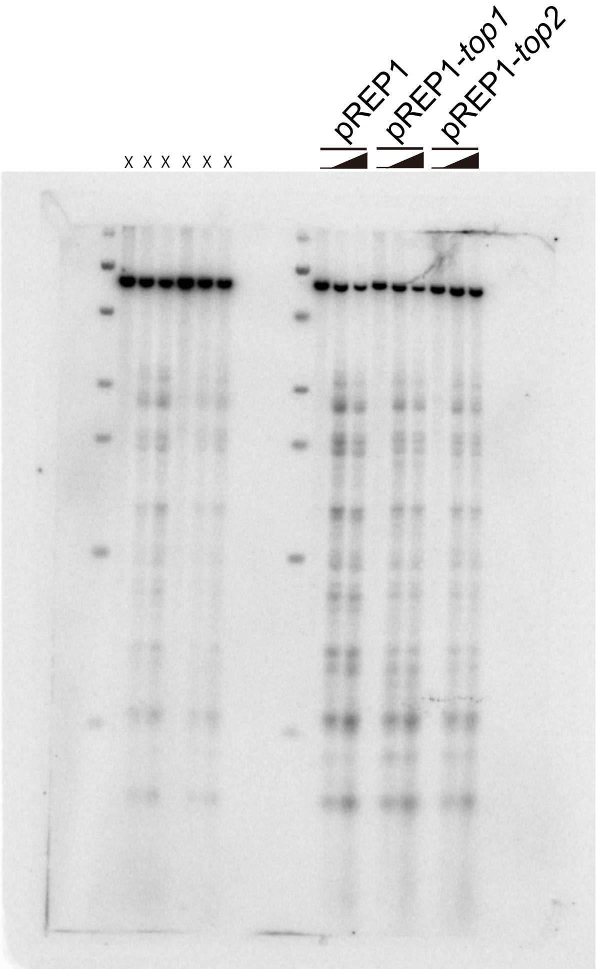

Supplement: S1 Raw images — (PDF) [file pone.0242348.s011.pdf]
